# Microbial perspective of multidisciplinary collaborative weight management approach: *Ruminococcus gnavus* may serve as a key target for weight loss

**DOI:** 10.1080/19490976.2024.2442038

**Published:** 2024-12-31

**Authors:** Sijia Zhao, Wenjun Wu, Wenyan Song, Qunyan Zhou, Haiyan Cheng, Shukun Deng, Jianxin Zhao, Hao Zhang, Wei Chen, Hongchao Wang

**Affiliations:** a State Key Laboratory of Food Science and Technology, Jiangnan University, Wuxi, Jiangsu, China; b School of Food Science and Technology, Jiangnan University, Wuxi, Jiangsu, China; c Jinshan Branch of Shanghai Sixth People’s Hospital, Shanghai, China; d The Affiliated Wuxi People’s Hospital of Nanjing Medical University, Wuxi People’s Hospital, Wuxi Medical Center, Nanjing Medical University; e National Engineering Research Center for Functional Food, Jiangnan University, Wuxi, Jiangsu, China; f Wuxi Translational Medicine Research Center and Jiangsu Translational Medicine Research Institute Wuxi Branch, Wuxi, Jiangsu, China

**Keywords:** Gut microbiota, obese, bile acid, *Ruminococcus gnavus*, machine learning

## Abstract

Changes in the gut microbiota are associated with obesity and may influence weight loss. We are currently implementing a sustained multidisciplinary collaborative weight management (MCWM) approach to weight loss. We report significant improvements in participant health status after 6 months, along with alterations in the structure, interactions, and metabolic functions of the microbiota. We observed an enrichment of the gut symbiont *Ruminococcus gnavus* in obese subjects, which was significantly correlated with clinical indicators and contributed significantly to random forest (RF) classification, and revealed its correlation with bile acid metabolism. Experimental results indicate that *R. gnavus* impairs weight loss in diet-modified obese mice by altering the gut microbiota structure and influencing bile acid metabolism. The findings of this study highlight the significant role of the gut microbiota in obese patients and establishes a more substantial scientific foundation for the prevention and management of obesity.

## 1. Introduction

Obesity, recognized as a global health challenge, is primarily characterized by the excessive accumulation of fat within the body, which is difficult to treat and is prone to recurrence. Obesity contributes to depression, impaired body image, inferiority complex, eating disorders, anxiety, stress, and reduced quality of life and also leads to imbalances in carbohydrate and lipid metabolism, fatty liver disease, increased risk of cardiovascular disease and cancer, and ultimately reduced life expectancy.^1,2^ Even a moderate (5–10%) reduction in body weight can significantly improve cardiovascular and metabolic functions.^3^ Current treatments for obesity primarily include lifestyle interventions,^4^ pharmacotherapy, and surgery. Dietary intervention is widely recognized as one of the most promising strategies for weight management. Although these interventions often yield short-term success, weight regain is inevitable.^5^ Therefore, more rational and effective weight-management strategies are required.

Energy-restricted diets effectively address the imbalance between energy consumption and expenditure, leading to weight loss, regulation of glucose homeostasis, and reduced inflammation.^6^ They are also effective in treating chronic diseases such as type 2 diabetes and dyslipidemia, with continuous energy restriction (CER) being the primary approach in numerous adult obesity guidelines.^7^ Although energy-restricted diets, including low-calorie diets (LCDs, recommended calorie intake of 800–1200 kcal/d) and very-low-calorie diets (VLCDs, recommended calorie intake of 500–800 kcal/d),^8^ along with restricted carbohydrate and fat intake, can effectively improve physiological parameters in obese individuals, maintaining adherence and overcoming compensatory eating remain significant challenges.^9,10^ To elucidate safer and more effective weight management strategies, studies have shown that a combination of moderate exercise and lifestyle modifications or other supplementary interventions is more effective than individual dietary adjustments in reducing weight and regulating metabolism.^11–13^

Current research indicates that the gut microbiota plays a crucial role in host energy metabolism.^14^ The gut microbiota and its metabolites, including short-chain fatty acids (SCFAs), indole derivatives, and trimethylamine N-oxides, influence various metabolic pathways, including energy balance, nutrient absorption, inflammatory pathways, appetite regulation, and metabolism.^15–18^ There is evidence that obese individuals experience changes in their gut microbiota after weight loss and that the degree of these changes is related to the intervention method. Weight-loss surgery increases the richness of the patient’s gut microbiota and the relative abundance of *Proteobacteria* (*Enterobacteriaceae*) and *Akkermansia* spp.^19^ A high-fat ketogenic diet results in the loss of bifidobacteria due to the antibacterial properties of the generated ketones.^20^ This indicates that the gut microbiota and its metabolites may be important mediators in response to different weight loss methods,^21,22^ emphasizing the potential benefits of the gut microbiota in regulating diet as a weight loss strategy. Therefore, analyzing the characteristics of gut microbiota using weight loss strategies can provide an opportunity to discover specific microorganisms associated with obesity.

Several classic animal studies have shown that germ-free (GF) mice fed a high-fat diet exhibit resistance to weight gain, yet those transplanted with feces from obese individuals become obese,^23,24^ suggesting that obesity phenotypes can be transmitted via the gut microbiota. Numerous systematic reviews have summarized the role of probiotics in weight loss in various animal models and clinical studies.^25–27^ Some studies have suggested that specific strains of *Lactobacillus* and *Bifidobacterium* exhibit optimal weight loss effects. Although the regulation of weight by probiotics remains controversial, the prevailing view is that probiotics (specifically bacterial strains) play a crucial role in weight management, improving metabolic functions, regulating blood sugar levels, reducing fat accumulation, and mitigating systemic inflammation. In addition, “next-generation probiotics,” such as *Akkermansia muciniphila* (*A. muciniphila*）^28^ and *Faecalibacterium prausnitzii* (*F. prausnitzii*),^29,30^ have significant effects on the regulation of metabolic disorders and insulin resistance, and clinical trials of these strains are eagerly anticipated. Overall, modifying the gut microbiota to enhance the host metabolic health is feasible.

In the present study, we implemented a 6-month multidisciplinary collaborative weight management (MCWM) approach focused on weight loss that combined a balanced diet with limited energy, daily exercise, and lifestyle adjustments. We recorded alterations in the characteristics of gut microbiota and metabolic status in obese subjects pre- and post-intervention. In addition, we used machine learning algorithms and other multidimensional methods to confirm that *R. gnavus* is a characteristic bacterium associated with obesity and has a regulatory role in weight loss in obese mice.

## 2. Methods

### 2.1. Participants and details of MCWM

#### 2.1.1. Subject recruitment

This study was conducted at Wuxi People’s Hospital, affiliated with Nanjing Medical University. Prior to recruitment, it was registered and approved by the hospital’s ethics committee (KYLLKS 2,019,020) and registered on the Chinese drug clinical trial website (ChiCTR1900022948). The Work Group on Obesity in China (WGOC) has established a reference standard for obesity among Chinese adults, defining obesity as a BMI greater than 28 kg/m^2.31^ This threshold is lower than the WHO’s global BMI standard of ≥30, but is more appropriate for the Chinese population due to higher body fat percentages and increased health risks at lower BMI levels.^32,33^

Inclusion criteria included individuals aged 18–65 years who comprehended and signed informed consent forms, with no gender restrictions. Participants were required to meet criteria for abdominal obesity, defined as a waist circumference of ≥90 cm for males and ≥85 cm for females, based on the International Diabetes Federation (IDF) metabolic syndrome definition, in conjunction with the standards of the Chinese Diabetes Society (CDS). Additionally, participants had to exhibit two or more metabolic abnormalities, including TG ≥ 1.7 mmol/L with or without treatment, HDL cholesterol levels <1.04 mmol/L or receiving relevant medical treatment, SBP ≥ 130 mmHg or DBP ≥ 85 mmHg or receiving relevant medical treatment, or a previous diagnosis of hypertension, and FBG ≥ 5.6 mmol/L or a previous diagnosis of Type 2 Diabetes.

Subjects were excluded if they met any of the following criteria: ① Secondary obesity patients, including those with conditions such as hypothyroidism, Cushing’s syndrome, or prolonged use of obesity-inducing medications; ② Patients with chronic diseases affecting organs such as the heart, brain, liver, and kidneys, requiring special diets and impacting exercise; ③ Patients with rheumatic and autoimmune disorders, hematological diseases, or a history of malignant tumors; ④ Pregnant or lactating women; ⑤ Individuals unable, unwilling, or noncompliant with research requirements, including lifestyle modification follow-up and subject obligations. Termination criteria included: ① Subject withdrawal of informed consent; ② Researcher determination that continuing the trial is harmful to the subject; ③ Subject development of additional illnesses after enrollment, rendering further participation in the study inappropriate as determined by the researchers.

#### 2.1.2. Details of MCWM

##### 2.1.2.1. Energy-limited balanced Diet

① Energy Intake: Calculated based on lean body mass: BMR (male and female) = 370 + (21.6 × lean body mass in kg), recommended energy intake = BMR × Activity Factor (Table S1) − 500 kcal. ② The energy supply ratio of diet: 40% carbohydrates, 25% protein, and 35% fat.

##### 2.1.2.2. Exercise plan

① Aerobic Exercise: The cardiopulmonary exercise test precisely determines the heart rate at the anaerobic threshold, denoted as HRAT. The mode of exercise is brisk walking, maintaining a heart rate of (HRAT ±10) beats per minute for 30–60 minutes per session (excluding warm-up and stretching), with a frequency of 5–7 sessions per week. The exercise routine includes warm-up, main workout, and stretching. ② Resistance Exercise: Conducted in 10 sets twice weekly, with 1-minute rest every 5 minutes. Exercises include hip bridges, plank supports, static wall squats, and 60-degree double straight-leg raises, each held for 30 seconds. Exercise heart rate monitoring is facilitated by wearing a Huawei Band 4 wristband, with patients instructed by the same rehabilitation physician.

##### 2.1.2.3. Lifestyle adjustments

Consume 1500–2000 ml of water daily, take one multivitamin tablet daily, and sleep before 11 pm.

##### 2.1.2.4. Drug therapy

The drug treatment plan was primarily formulated by the attending physicians based on each patient’s specific condition, while also considering the patient’s preferences.

Glucose-lowering drug administration: Metformin therapy was initiated at 1.0 g/day and titrated up to the patient’s maximum tolerated dose of 2.0 g/day within two weeks, with the final dose sustained until the end of the study. For patients unable to tolerate the maximum dose, a minimum of 1.0 g/day was maintained until study completion. Liraglutide was administered through subcutaneous injection, starting at 0.6 mg/day. The dosage was escalated weekly by 0.6 mg/day until reaching a maximum of 1.8 mg/day, which was sustained throughout the study period.

Antihypertensive drug administration: Among the 47 patients, 6 had a prior diagnosis of hypertension and had been on stable antihypertensive therapy for over three months prior to enrollment. Their initial antihypertensive regimens and dosages were continued throughout the study.

Participants who met the inclusion criteria undertook a 6-month MCWM Approach under the guidance of a physician. A WeChat group was created to maintain close contact with patients, providing communication and guidance. Patients daily report their diet type, food quantity, weight data, and any discomfort through pictures and text messages in the group. The dietary questionnaire recorded the patients’ meal times, dietary types, and eating habits. Throughout the program implementation, the nutritionist will daily monitor the WeChat group. In case of issues, the nutritionist will address them, decide on solutions, and keep records. Outpatient visits occur monthly to assess the program’s progress.

#### 2.1.3. Oral glucose tolerance test and blood sample collection

Before and after the MCWM intervention, participating patients after fasting for at least 8 hours for intravenous blood collection, which was required for subsequent testing of serum biochemical parameters. Following this, participants were instructed to rapidly consume 75 g anhydrous glucose dissolved in 250–300 mL of water within 5–10 minutes. Two hours later, venous blood samples were collected again for the measurement of 2-hour blood glucose and 2-hour insulin levels. The collected blood samples were immediately tested, while any remaining samples were swiftly stored at −80°C.

#### 2.1.4. Anthropometric assessment and body composition assessment

Record the subjects’ height, weight, waist circumference, and hip circumference, then calculate their BMI using the formula: weight (kg) divided by the square of height (m^2^). Additionally, compute the waist-to-hip ratio (WHR), defined as the ratio of waist to hip circumferences. Measure systolic and diastolic blood pressure (SBP and DBP) using a medical electronic blood pressure monitor (OMRON, HBP-1100 U). Body composition was measured using a body composition analyzer, including skeletal muscle mass, body fat mass, and body fat percentage. All parameters were recorded both before MCWM and six months after the intervention.

#### 2.1.5. Fecal sample collection and storage

Fecal samples were collected in sterile tubes to investigate changes in gut microbiota pre- and post- MCWM. The first fecal sample was obtained either the day before initiating MCWM or on the morning of its commencement. The second fecal sample was collected at the end of MCWM.

### 2.2. Animals and experimental design

The animal experimental protocol (JN.No20221215c2280710[536]) was reviewed and approved by the Animal Ethics Committee of Jiangnan University. C57BL/6J male mice, aged 4 weeks and weighing approximately 18–20 g, were procured from Beijing Vital River Laboratory Animal Technology Co., Ltd. and housed in the animal experimental center of Jiangnan University under specific pathogen-free (SPF) conditions (20–26°C, 40%–70% relative humidity, 12 h light-dark cycle). Model-building methods were derived from literature references and adjusted as needed. During the acclimation period, mice had ad libitum access to water and were fed a standard diet. After one week of acclimation, six mice were randomly assigned to the blank control group and fed a low-fat control diet, while the remaining mice were fed a high-fat diet (HFD: 60% fat, 19% protein, 21% carbohydrates). At the end of the 12th week, 18 mice weighing 20%–25% more than the blank control group were considered successful models and randomly divided into three groups: HFD, LFD, and LFD-Rg. Three mice were housed per cage, with two cages forming a group. After the start of the intervention period, every two weeks, the six mice from the same group were randomly redistributed into two new cages to minimize the potential impact of cage effects. The weight loss period began thereafter, during which the HFD group continued on a high-fat diet while the other two groups switched to a low-fat control diet. Additionally, the LFD-Rg group received a *R. gnavus* bacterial solution (2 × 10^9^ CFU) for eight weeks. Oral glucose tolerance testing (OGTT) was performed at the end of week 20. Fecal samples from each group were collected and stored at −80°C until use. Weekly body weight measurements were recorded throughout the experiment.

### 2.3. Source of strains and cultivation conditions

*Ruminococcus gnavus* FWXVP1Y79 was preserved at the JiangNan University Culture Collection of Food Microorganisms were cultured with BHI medium containing 0.5% cysteine hydrochloride at 37°C for 24 hours and then activated. The culture was continuously activated for 3 generations under the same conditions in the medium with a 4% inoculation amount, and the *R. gnavus* strain (4th generation) was collected. Centrifuge the *R. gnavus* bacterial solution at 8000 r/min for 10 minutes, wash the *R. gnavus* bacterial cells once with 0.9% physiological saline (containing 0.5% cysteine hydrochloride) pre-cooled to 4°C, and finally collect the bacterial cells by centrifugation under the same conditions. The obtained bacterial cells were resuspended with pre cooled 30% glycerol, gradient diluted and counted on a plate, stored in a −80°C refrigerator for future use, washed with sterile physiological saline to remove glycerol, and diluted to a viable bacterial count of 1 × 10^10^ CFU/mL.

### 2.4. Metagenomic sequencing

94 feces from the population were subjected to metagenomic sequencing using the DNBSEQ-T7 platform PE150 of Beijing Nuohe Zhiyuan Technology Co., Ltd. Quality control of the obtained raw sequencing data is carried out as follows: 1. Splicing: Utilize the Trimmatic tool to align obtained reads with the sequenced splice sequence, thereby eliminating any potential residual splice sequences; 2. Remove low-quality bases: Employ Trimmatic to compute the average quality score of bases, starting from the 5’ end with a 4 bp window. If the average value falls below 30, truncate the read from that position; Discard any reads with a length less than 60 bp from both ends; 3. Remove host: Utilize BWA tools to align reads against the human genome (Homo sapiens, UCSC hg38) and filter out host reads. Employ MetaPhlAn2 to annotate species of gene sequences post-quality control. Merge species abundance data for each sample using MetaPhlAn2, thereby generating a table of species abundance across multiple samples. Subsequently, 15 mouse fecal samples underwent analogous procedures, substituting the human genome with the mouse genome (UCSC GRCm39) for comparison, post host removal.

### 2.5. Non-targeted metabolomic detection

#### 2.5.1. Metabolites extraction of serum samples

100 μL of sample was taken, mixed with 400 μL of extraction solution (MeOH:ACN, 1:1 (v/v)), the extraction solution contain deuterated internal standards, the mixed solution were vortexed for 30 s, sonicated for 10 min in 4°C water bath, and incubated for 1 h at −40°C to precipitate proteins. Then, the samples were centrifuged at 12,000 rpm (RCF = 13800(×g), *R* = 8.6 cm) for 15 min at 4°C. The supernatant was transferred to a fresh glass vial for analysis. The quality control (QC) sample was prepared by mixing an equal aliquot of the supernatant of samples.

#### 2.5.2. Metabolites extraction of fecal sample

25 mg fecal sample were taken, mixed with 500 μL of extraction solution (MeOH:ACN:H2O, 2:2:1 (v/v)), the extraction solution contain deuterated internal standards, the mixed solution were vortexed for 30 s. Add 2 homogenization beads and homogenize for 4 min (35 hz), then transferred to an ice-water bath to sonicate for 5 min (Repeat 3 times). The samples were then allowed to thaw at room temperature and vortexed for 30 s. This freeze – thaw cycle was repeated three times. Then the samples were sonicated for 10 min in 4°C water bath, and incubated for 1 h at −40°C to precipitate proteins. The samples were centrifuged at 12,000 rpm (RCF = 13800(×g), *R* = 8.6 cm) for 15 min at 4°C. The supernatant was transferred to a fresh glass vial for analysis. The quality control (QC) sample was prepared by mixing an equal aliquot of the supernatant of samples.

#### 2.5.3. LC-MS/MS analysis

For polar metabolites, LC-MS/MS analyses were performed using an UHPLC system (Vanquish, Thermo Fisher Scientific) with a Waters ACQUITY UPLC BEH Amide (2.1 mm × 50 mm, 1.7 μm) coupled to Orbitrap Exploris 120 mass spectrometer (Orbitrap MS, Thermo). The mobile phase consisted of 25 mmol/L ammonium acetate and 25 mmol/L ammonia hydroxide in water (pH = 9.75) (A) and acetonitrile (B). The auto-sampler temperature was 4°C, and the injection volume was 2 μL. The Orbitrap Exploris 120 mass spectrometer was used for its ability to acquire MS/MS spectra on information-dependent acquisition (IDA) mode in the control of the acquisition software (Xcalibur, Thermo). In this mode, the acquisition software continuously evaluates the full scan MS spectrum. The ESI source conditions were set as following: sheath gas flow rate as 50 Arb, Aux gas flow rate as 15 Arb, capillary temperature 320°C, full MS resolution as 60,000, MS/MS resolution as 15,000, collision energy: SNCE 20/30/40, spray voltage as 3.8 kV (positive) or −3.4 kV (negative), respectively.

### 2.6. Bioinformatics analysis

The data undergo dimensionality reduction using linear discriminant analysis effect size (LEfSe) to identify main influencing factors and evaluate the impact of different species. A significance level of 0.05 is set for intergroup differences. The linear discriminant analysis (LDA) threshold is set to 2, identifying LDA values exceeding 2 as differential species.

Machine learning models were employed to assess classification accuracy and identify key bacterial genera. Seven classification models were used, including k-Nearest Neighbor (kNN), Support Vector Machine (SVM), Decision Tree (DT), Random Forest (RF), Gradient Boosting Regression Tree (GB), Extreme Gradient Boosting (XGB), and LightGBM (LGB), along with Extreme Gradient Boosting Tree (XGB and XGBRF) and Light Gradient Boosting (LGB) models. These models were implemented using the scikit-learn Python package. To mitigate model overfitting and ensure accurate performance assessment, a 10-fold cross-validation with five replications was conducted. The number of decision trees in the random forest model (n_estimators) was set to 400, with a maximum tree depth (max_depth) limited to 15. The minimum number of samples required at a leaf node (min_samples_leaf) was set to 2, and the minimum number of samples required to split a node (min_samples_split) was also set to 2. The feature importance was assessed based on Gini impurity.^34^ The performance of the random forest model was evaluated using the area under the ROC curve (AUC), accuracy, precision, out-of-bag (OOB) error, and the confusion matrix.

Species with relative abundances present in at least 10% of the samples were selected to construct a co- occurrence network. The screened bacterial abundance table served as the original dataset, and correlations were computed using the Rcorr function of the Hmisc package in R. Correlation relationships with absolute correlation coefficients exceeding 0.6 and *p* values below 0.05 were considered significant. Gephi 0.9.7 software was utilized to construct a coexistence network of gut microbiota within the population.

We performed a MaAsLin correlation analysis to examine the associations between differential species and physiological indicators in obese subjects before and after the intervention, while controlling for gender, age, and medication treatment status as potential confounders.

### 2.7. Method for indicator measurement

#### 2.7.1. Assessment of biochemical parameters in human serum

A fully automated biochemical analyzer is used to assess various indicators including alanine aminotransferase (ALT), albumin (ALB), fasting blood glucose (FBG), glycated hemoglobin (HbA1c), aspartate aminotransferase (AST), urea (BUN), creatinine (CREA), total cholesterol (TC), triglycerides (TG), high-density lipoprotein cholesterol (HDL-C), low-density lipoprotein cholesterol (LDL-C), alkaline phosphatase (ALP), and serum insulin (INS) levels in the supernatant of vacuum blood collection vessels. Samples are promptly stored at −80°C.

#### 2.7.2. Animal serum biochemical assays

Prior to the OGTT, mice were fasted for 12 hours overnight. Blood was collected from the tip of the tail vein to measure fasting blood glucose levels. Subsequently, mice received an oral administration of 2 g/kg·bw 25% glucose, and blood glucose levels were measured at 30, 60, 90, and 120 minutes using a glucose meter (Accu Check; Roche, Basel, Switzerland). The area under the curve (AUC) was calculated using GraphPad Prism 9.5. Serum levels of AST, ALP, TC, TG, HDL-C, and LDL-C were measured using reagent kits obtained from Nanjing Jiancheng Biotechnology Co., Ltd.

#### 2.7.3. Histopathological analysis

Harvest liver and epididymal fat from mice, then fix them in 4% paraformaldehyde solution. Following washing, dehydration, transparency, waxing, embedding, and sectioning, 3 mm thick paraffin sections were stained with hematoxylin and eosin (H&E). Examine liver and epididymal adipose tissue sections using digital slide scanners at magnifications of 200 × and 400 ×, respectively (Pannoramic MIDI II, 3DHISTECH, Budapest, Hungary).

### 2.8. Statistical analysis methods

SPSS 26.0 (IBM Corporation, Somers, NY, USA) was utilized for analysis of covariance (ANCOVA), controlling for covariates (age, gender) and assessing significant differences among groups. Data are presented as mean ± standard error of the mean (SEM). Significance is denoted as *p < 0.05, **p < 0.01, and ***p < 0.001. Normality was assessed using the Shapiro–Wilk test; data with *p* > 0.05 were considered to have a normal distribution.

## 3. Results

### 3.1. MCWM alters the anthropometric characteristics and biochemical parameters of obese subjects

Forty-seven participants (24 females and 23 males) completed the study, with an average age of 30.00 ± 7.69 years (Table 1). Weight loss exceeding 5% of the baseline was considered effective, with 36 participants successfully losing weight. The success rate of MCWM was 76.60%. At the beginning of the study, the average body mass index (BMI) of the participants was 35.03 ± 4.67 kg/m^2^. After 6 months of treatment, both the average weight loss and BMI of the obese participants were significantly reduced (*p* < 0.001). There were also significant decreases in WC and HC. We observed a decline in SBP and DBP in the obese subjects after the intervention. Following the intervention, TG and TC concentrations decreased. The improvement in HDL-C levels following the intervention was significantly greater in female patients compared to male patients. The levels of AST, and ALP, which indicate liver damage, significantly decreased, suggesting that the MCWM mitigated liver injury to some extent. Following the intervention, both fasting and postprandial blood glucose and insulin levels declined, indicating that MCWM could ameliorate abnormal blood glucose and insulin regulation. Although there was an increase in the concentrations of CREA and BUN, which are markers of kidney damage, they remained within the normal ranges. Interestingly, we observed a decrease in total bile acid levels in the obese subjects(Table 2). Research has shown a correlation between the blood levels of total bile acids and obesity,^35^ suggesting a regulatory role in fat metabolism and energy balance. Additionally, the body composition of obese participants changed following the intervention. There was a significant reduction in body fat and body fat percentage, although skeletal muscle mass and basal metabolic rate also showed slight decreases (Table S2). Consequently, changes in these levels reflect the therapeutic effects of MCWM on obesity. These findings suggest that MCWM improve the anthropometric characteristics and clinical biochemical indicators in obese individuals.

**Table 1.** Basic information of subjects.

Index	Baseline information (Mean±SEM)
Age (years)	30.00±7.69
Gender (male:female)	23M:24F
BMI (kg/m^2^)	35.03±4.67
Medication administrition	Number of subjects
Metformin	6
Liraglutide	8
Both Metformin and Liraglutide	6
No glucose-regulating medication	27

**Table 2.** Clinical parameters of subjects.

Index	Pre intervention	Post intervention	*P*	Change	Change rate
BW [kg]	100.723±17.649	88.843±18.370	<0.001	−11.270±8.543	−11.369±8.642%
BMI [kg/m^2^]	35.166±4.717	31.036±5.236	<0.001	−3.991±3.154	−11.365±8.629%
WC (cm)	109.113±11.157	97.111±14.089	<0.001	−11.639±9.320	−10.744±8.488%
HC (cm)	113.755±9.159	105.913±11.238	<0.001	−7.750±8.200	−6.747±7.008%
WHR	0.960±0.066	0.915±0.066	<0.001	−0.042±0.045	−4.341±4.561%
SBP [mmHg]	134.174±15.261	122.239±13.874	<0.001	−11.711±13.614	−8.280±9.250%
DBP [mmHg]	79.913±10.670	70.543±9.145	<0.001	−9.156±9.210	−10.807±10.325%
ALT [U/L]	70.826±53.015	39.093±51.471	0.090	−31.700±69.695	−20.129±130.713%
AST [U/L]	43.657±26.439	25.152±16.556	0.006	−18.520±26.447	−28.897±54.687%
ALP [U/L]	84.700±24.006	52.583±13.804	<0.001	−32.633±26.471	−33.958±22.885%
ALB [g/L]	45.411±5.228	72.589±47.915	0.002	27.137±47.515	59.845±99.505%
BA [µmol/L]	3.950±2.770	2.551±1.236	0.025	−1.468±2.821	6.511±125.479%
GGT [U/L]	51.636±28.403	47.920±77.149	0.485	−3.730±81.129	43.024±326.832%
CREA [µmol/L]	66.813±16.569	70.187±14.970	<0.001	3.346±6.892	6.521±11.792%
BUN [mmol/L]	4.550±1.325	5.133±1.117	<0.001	0.593±1.188	20.965±42.379%
UA [µmol/L]	433.315±131.310	421.193±106.124	0.030	−12.28±99.702	11.563±97.523%
TG [mmol/L]	2.359±1.243	1.688±0.963	0.039	−0.680±1.201	−21.500±38.062%
TC [mmol/L]	5.249±1.054	5.005±0.936	0.008	−0.267±0.941	−3.420±17.116%
LDL-C [mmol/L]	3.239±0.729	2.992±0.752	0.079	−0.259±0.641	−6.884±19.926%
HDL-C(male) [mmol/L]	0.961±0.156	1.012±0.145	0.263	0.040±0.117	5.020±12.641%
HDL-C(female) [mmol/L]	1.076±0.170	1.137±0.191	0.038	0.061±0.160	6.584±14.951%
HbAlc [%]	6.664±1.952	5.373±0.386	<0.001	−1.318±1.833	−15.317±16.325%
FBG [mmol/L]	6.491±2.780	5.156±0.614	<0.001	−1.373±2.719	6.114±148.504%
BG2h [mmol/L]	10.100±5.180	6.968±1.856	<0.001	−3.213±4.650	−22.080±25.060%
INS0h [μIU/ml]	30.317±17.869	19.815±16.152	0.004	−9.804±15.420	−25.086±54.612%
INS2h [μIU/ml]	134.216±106.711	84.784±88.466	0.013	−49.140±88.265	−20.593±53.881%

BW: body weight; BMI: body mass index; WC: waist circumference; HC: Hip circumference; WHR: Waist to hip ratio; SBP: systolic blood pressure; DBP: diastolic blood pressure; ALT: alanine transferase; AST: aspartate transaminase; ALP: alkaline phosphatase; BA: bile acid; GGT: gamma-glutamyl transpeptidase; CREA: creatinine; BUN: blood urea nitrogen; UA: uric acid; TG: triglyceride; TC: total cholesterol; LDL -C: low-density lipoprotein cholesterol; HDL-C: high density liptein cholesterol; HbA1c: hemoglobin Alc; FBG: fasting blood glucose; BG2h: 2-hour blood glucose; INS0h: Fasting insulin; INS2h: 2-hour insulin. The change and change rate were calculated based on the difference between the post- and pre-intervention values. P-values were derived from analysis of covariance (ANCOVA), with adjustments for gender and age as covariates.

Non-diabetic patients appeared to have a more pronounced response to MCWC, particularly in terms of liver function and glycemic parameters. In diabetic patients, there were no significant changes in ALT and AST levels before and after the intervention, and while blood glucose was controlled, the changes were not statistically significant. For non-diabetic patients, significant improvements were observed in both liver function and glycemic regulation.

### 3.2. MCWM alters the gut microbiome and metabolic pathways in obese subjects

#### 3.2.1. MCWM alters the composition of gut microbiota in obese subjects

**Figure 1.**
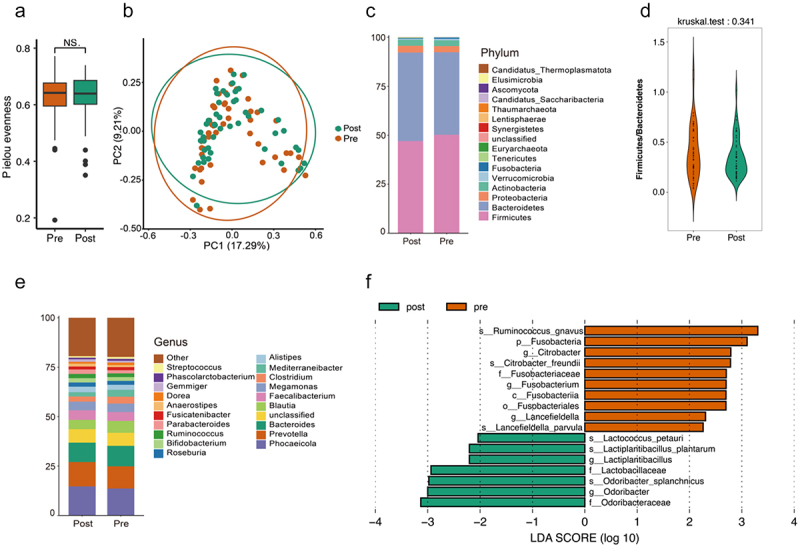
Differences in the gut microbiome before and after the MCWM. a: microbiome alpha diversity based on Pielou’s evenness (*p* <0.05); b: principal coordinate analysis (PCoA) plot illustrating the beta diversity of microbiome samples based on weighted bray distance; c: stacked bar chart displaying the relative abundance of bacterial phyla in both groups; d: differences in gut microbiota Firmicutes/Bacteroidetes before and after MCWM; e: stacked bar chart representing the relative abundance of bacterial genera in each group; f: linear discriminant analysis (LDA) effect size (LEfSe) analysis highlighting significantly different bacterial taxa between the pre- and post-mcwm.

The Pielou’s evenness, Shannon index and Simpson index were used to illustrate the differences in the gut microbiome before and after the MCWM (Figure 1a, Figure S1 B and C). These indices reflect the richness of the gut microbiome, the even distribution of microbial abundance, and overall diversity. There were no significant differences in the Shannon index, Simpson index, or Pielou’s evenness before and after the MCWM. Beta diversity measurements were used to capture the compositional variations among the samples. bray-curtis distance analysis was employed to compute the beta diversity between the two groups before and after the intervention. There were no significant differences in the beta diversity of the gut microbiota before and after the intervention (Figure 1b).

Although there were significant individual differences in the composition and structure of the gut microbiota among obese subjects before and after the intervention (Figure S1 A), we observed that the abundance of Firmicutes decreased and that of Bacteroidetes increased in most subjects after the intervention. The ratio of Firmicutes to Bacteroidetes has been confirmed to be closely related to obesity, with a significant increase in the abundance ratio of Firmicutes to Bacteroidetes in the gut of obese individuals. Firmicutes/Bacteroidetes was observed to decrease in obese subjects after MCWM, although there was no significant difference (Figure 1d). At the genus level, the relative abundance of *Phocaeicola*, *Prevotella*, *Bacteroides*, *Alistipes*, *Bifidobacterium*, *Faecalibacterium*, *Paraacteroides*, and *Ruminococcus* increased (Figure 1d). The relative abundances of *Blautia*, *Megamonas*, *Dorea*, and *Escherichia* decreased. Furthermore, at the species level, LEfSe analysis shows that after MCWM, *R. gnavus*, *Citrobacter freundii*, and *Lancefieldella parvula* significantly decreased. There was a significant increase in *Lactiplantibacillus plantarum*, *Lactococcus petauri*, and *Odoribacter splanchnicus (O. splanchnicus)* (Figure 1f).

In addition to analyzing the gut microbiota structure of all obese subjects, we also compared the microbiota structure of diabetic and non-diabetic subjects before and after the intervention (Figure S2). We found that the baseline Simpson index differed between diabetic and non-diabetic subjects, but this difference disappeared after the intervention. Compared to non-diabetic subjects, diabetic subjects had lower relative abundances of *Bacteroides* and *Parabacteroides*. After the intervention, diabetic subjects showed an increase in the relative abundances of *Faecalibacterium* and *Ruminococcus*, while *Blautia* decreased. Overall, the richness and evenness of the gut microbiota in diabetic and non-diabetic subjects did not change significantly before and after the intervention, but the abundance of certain genera did vary. Additionally, studies have shown that antidiabetic drugs, including liraglutide and metformin, alter the gut microbiota.^36,37^ However, in this study, no significant effect of antidiabetic drugs on the gut microbiota was observed (Figure S 3–5). Furthermore, when drug usage status was analyzed as a confounding factor, the impact of medication on gut microbiota alpha diversity approached significance but remained statistically non-significant (Table S4). Similarly, in this study, no effect of gender on the gut microbiota structure was observed before and after the MCWM intervention (Figure S6).

#### 3.2.2. Machine learning identifies key gut microbiota before and after MCWM

**Figure 2.**
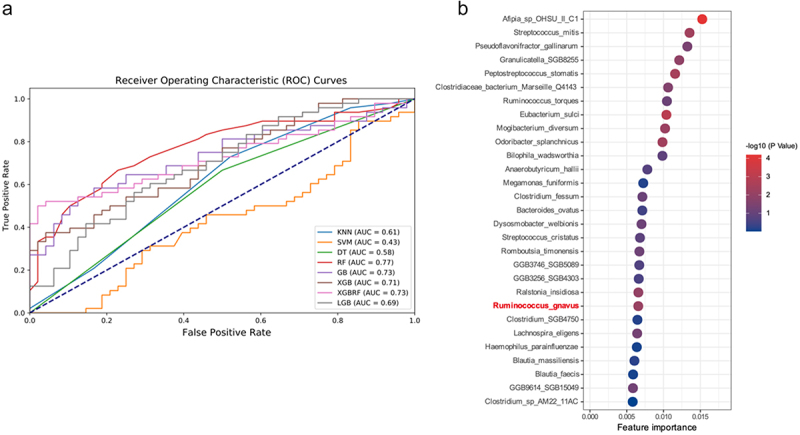
Predictive analysis of gut microbiome data. a: receiver operating characteristic (ROC) curves for different machine learning models evaluating the classification performance of gut microbiome features. b: top 30 species ranked by feature importance from the random forest classification algorithm, with colors indicating the significance level (-log10 of P-values) for each species.

Machine learning algorithms was conducted on the metagenomic species-level information to study the key microbial features affected by multidisciplinary joint interventions. Eight machine learning classifier methods were employed to differentiate the species composition of the microbiota before and after intervention. The ROC curves and AUCs for each classifier are shown in Figure 2. The random forest (RF) classifier exhibited superior performance, achieving an AUC of 0.77 (Figure 2A). The other performance parameters of RF are reported in Supplementary Table 5. Subsequently, the top 30 gut microorganisms were identified based on the importance of their RF features. Notably, LEfSe analysis combined with the Wilcoxon paired rank sum test revealed a statistically significant variation in the abundance of *O. splanchnicus* and *R. gnavus* pre- and post-MCWM treatment. Similarly, the feature importance of *O. splanchnicus* and *R. gnavus* in the random forest (RF) algorithm ranked 10th and 22nd, respectively, among 1387 gut microbiota species identified in obese patients, significantly influencing the RF classification outcomes. Our findings revealed a significant upward trend in *O. splanchnicus* abundance following the intervention. Prior research has indicated that *O. splanchnicus*, as a producer of SCFAs,^38^ exhibits a negative correlation with host fat content.^39^ In addition, following a low-carbohydrate, high-fat, weight loss diet rich in fiber, the abundance of *O. splanchnicus* increased.^40^ The abundance of *R. gnavus* significantly decreased following the intervention, corroborating the positive correlation between *R. gnavus* and BMI, along with its strong association with fat mass.^41^ These findings highlight the potential significance of *O. splanchnicus* and *R. gnavus* in the regulation of the host energy metabolism during multidisciplinary interventions.

#### 3.2.3. MCWM alters the gut microbiota co-occurrence network in obese subjects

**Figure 3.**
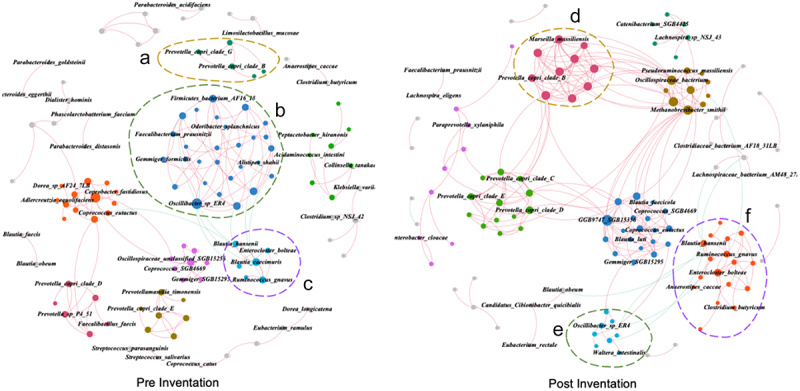
Effect of MCWM on gut microbiome co-occurrence networks. The co-occurrence networks illustrate microbial interactions before (left) and after (right) the intervention. Each node represents a microbial species, while edges indicate co-occurrence relationships between species. Clusters labeled a, b, c (pre-intervention) and d, e, f (post-intervention) highlight key microbial communities affected by the intervention.

To assess the responsiveness of gut microbiota interactions in obese subjects to the MCWM, we constructed a co-occurrence network using the following criteria: strong correlation (| r |>0.6) and *p* < 0.01. Before the intervention, 312 species exhibited 457 positive correlations and eight significant negative correlations (Table 3). However, post-intervention, 347 species showed 610 positive and eight significant negative correlations (Table 3). The topological characteristics of each node within the network were computed. A comparison of the two networks revealed that the average clustering coefficient in the species-level co-occurrence network increased from 0.621 to 0.691 post-intervention, suggesting a shift toward more intricate microbial network interactions post-intervention. Using the modular algorithm in Gephi 0.9.7 software, we clustered nodes based on their tight connectivity, distinguishing and coloring nodes by category. The number of modular clusters increased from 200 pre-intervention to 216 post-intervention, with two primary sub-clusters of more than ten nodes identified in the pre-intervention network. In the post-intervention network, five sub-clusters were identified and the number of sub-clusters with more than ten nodes increased. In the post-network, five major sub-clusters with more than ten nodes were identified (Figure 3), and the number of sub-clusters containing more than ten nodes increased. These findings suggest that, following the intervention, the interactions between the gut microbiota species within each subgroup became more complex and concentrated. The relationships among the different subgroups also changed. Before the intervention, several major subgroups were relatively independent; however, post-intervention, the interactions among these primary subgroups became more intimate. The negative correlation between the *R. gnavus* and *Oscillibacter sp* ER4 modules diminished, whereas the negative correlation with the *Lachnospiraceae bacterium* AM48 27BH module intensified. Notably, the gut microbiota types within each subgroup changed after the intervention. *F. prausnitzii* was reclassified from the originally assigned *Oscillibacter sp* ER4 module, accompanied by an increased positive correlation with *Lachnospira eligens*. The *Prevotella copri clade B* module expanded from only five nodes to a significant module with 15 nodes. These results implied that the subjects’ microbial symbiotic networks evolved toward healthier and more intricate states.

**Table 3.** Network topology structure parameters.

Parameters	Pre intervention	Post intervention	Δ(|Post–Pre|)
Number of edges	465.000	618.000	153.000
Number of positive edges	457.000	610.000	153.000
Number of negative edges	8.000	8.000	0.000
Number of vertices	312.000	347.000	35.000
Average degree	2.981	3.562	0.581
Average path length	5.217	5.453	0.236
Diameter	12.000	16.000	4.000
Average clustering coefficient	0.621	0.691	0.070
Modularity	0.910	0.706	0.204
Number of modularity	200.000	216.000	16.000

Comparison of gut microbiome co-occurrence network parameters pre- and post-MCWM. The changes in each parameter (Δ) indicate alterations in the network structure following the intervention.

#### 3.2.4. MCWM alters the metabolic pathways of gut microbiota

**Figure 4.**
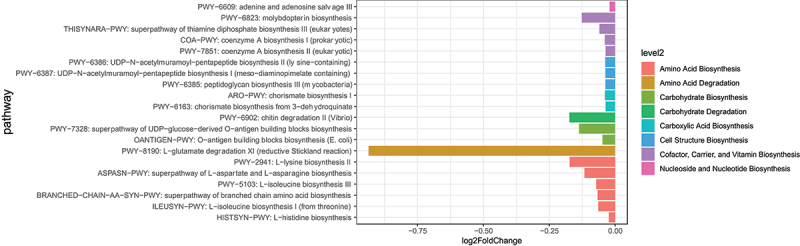
Differential analysis of microbial metabolic pathways. The bar chart displays the log2-fold changes in various metabolic pathways between the pre- and post- MCWM. Pathways are categorized by their functional levels (level 2), including amino acid biosynthesis, carbohydrate degradation, cell structure biosynthesis, and others, indicated by different colors. The length of each bar represents the magnitude of change in the pathway’s abundance following the intervention.

Analysis of the differential metabolic pathways in the gut microbiota revealed that, before and after MCWM, 75 distinct metabolic pathways were identified in the gut microbiota of obese subjects (*p* < 0.05). Figure 4 shows the top 20 pathways ranked by differential expression. The results indicated that the abundances of the following metabolic pathways decreased post-intervention. These included amino acid biosynthesis, amino acid degradation, carbohydrate biosynthesis, carbohydrate degradation, carboxylic acid biosynthesis, cell structure biosynthesis, cofactors, carriers, vitamin biosynthesis, and nucleoside and nucleotide biosynthesis.

### 3.3. MCWM alters metabolic status of obese subjects

To assess the effects of MCWM on the metabolic profiles of the participants, we performed untargeted metabolomic analyses of the serum and fecal samples before and after the intervention. We identified 10,224 and 11,405 metabolites in the serum and fecal samples, respectively. Figure S7 A illustrates the proportion of metabolites categorized under each superclass in both the serum and fecal samples. In serum samples, lipids and lipid-like molecules accounted for the largest proportion at 20.36%, whereas in fecal samples, organoheterocyclic compounds represented the largest proportion at 17.66%.

**Figure 5.**
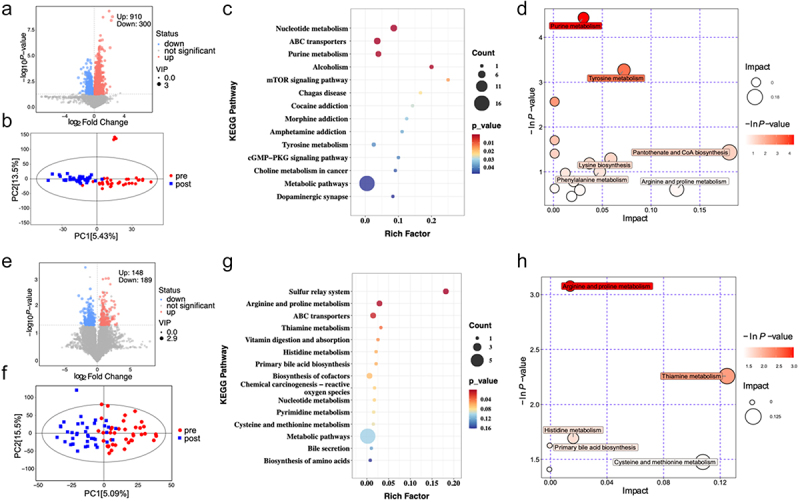
Differences in serum (a-d) and gut microbiota (e-h) metabolites of subjects before and after MCWM intervention. e, e: volcano plots highlighting the differential metabolites between pre- and post-mcwm. b, f: score scatter plots of the orthogonal partial least squares discriminant analysis (OPLS-DA) model for group separation. c, g: KEGG enrichment analysis of the identified differential metabolites. d, h: metabolic pathway analysis displaying the impact and significance of each pathway. The statistical comparison is conducted using the post- vs pre- MCWM.

To identify potential biomarkers for population differentiation, we employed orthogonal projections for a latent structure discriminant analysis (OPLS-DA). We focused on variables exhibiting a VIP score > 1 and a *p*-value < 0.05 in the first principal component. The results indicated that between the pre- and post-MCWM groups, 1,210 metabolites differed significantly in the serum samples (910 upregulated and 300 downregulated) and 337 differed significantly in the feces samples (148 upregulated and 189 downregulated). KEGG enrichment analysis of differential metabolites revealed significant pathways. The accompanying figure illustrates the top 15 pathways for differential metabolite enrichment before and after MCWM, with Metabolic pathways, nucleotide metabolism, and ABC transporters enriched in both the serum and fecal differential metabolites. Pathway analysis demonstrated the significance of pathway enrichment and impact factor size in the topological analysis. Figure 5 indicates that MCWM significantly impacted pathways like Purine metabolism in the subjects, as well as pathways involving gut microbiota such as Arginine and Proline metabolism. These findings demonstrate that MCWM can modify the metabolic conditions of both subjects and their gut microbiota.

### 3.4. Multiomics reveals the remodeling of gut microenvironment after weight loss

#### 3.4.1. R. gnavus *is closely associated with anthropometric parameters of obesity*

**Figure 6.**
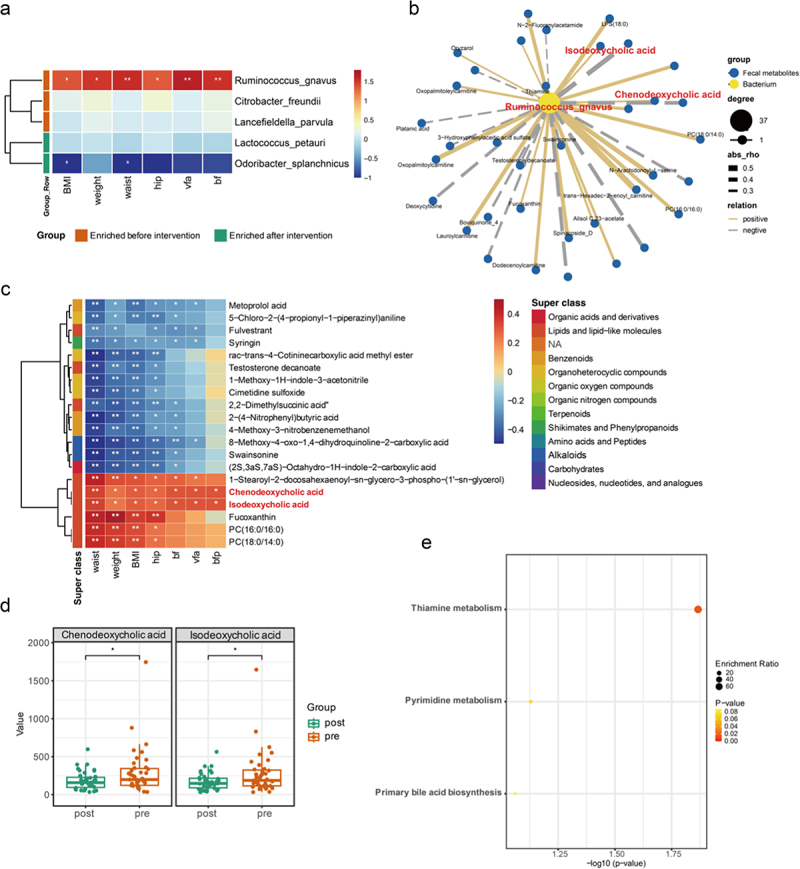
Multiomics reveals gut microenvironment remodeling after MCWM. A: the MaAsLin correlation between differential gut microbiota and obesity-related anthropometric measures; B: correlation between *R. gnavus* and differential metabolites in feces; C: the correlation between fecal differential metabolites associated with *R. gnavus* and obesity-related anthropometric measures; D: differences between two types of bile acids before and after MCWM; E: KEGG pathway enrichment analysis of fecal differential metabolites associated with *R. gnavus.*

MaAsLin correlation analysis was conducted on the anthropometric parameters related to obesity and the differential gut microbiota identified using LEfSe analysis in obese subjects before and after MCWM. Following the adjustment for potential confounders such as gender, age, and medication administration, *O. splanchnicus* and *Lactococcus petauri* were identified as inversely associated with obesity-related parameters. Importantly, *O. splanchnicus* was found to have a pronounced and statistically significant negative association with BMI and waist circumference (Figure 6). Notably, *R. gnavus* was significantly positively correlated with all six obesity-related parameters. These results suggest a close association between *R. gnavus* and phenotypic symptoms in obese patients.

#### 3.4.2. R. gnavus *affects bile acid metabolism during MCWM weight loss process*

Previous differential analysis, along with results from the random forest (RF) algorithm, suggest that *R. gnavus* could have a pivotal role in MCWM and is strongly associated with the phenotypic manifestations observed in obese patients. Therefore, we investigated whether *R. gnavus* is involved in the regulation of host metabolism. Association analysis between *R. gnavus* and differential metabolites in the serum and feces showed that there were 57 and 63 correlations (| r |>0.1) between *R. gnavus* and differential metabolites in the serum and feces, respectively. Serum metabolites showed 47 negative correlations with *R. gnavus* and 10 positive correlations, 29 positive correlations, and 34 negative correlations with fecal metabolites. Among the top 20 substances related to serum metabolites, six were organoheterocyclic compounds. Significant correlations between *R. gnavus* and differential metabolites in the serum and feces are shown in Figure 6 B and figure S7 B, respectively. Among the differential metabolites in the serum, eight substances were significantly correlated with *R. gnavus*, of which two substances belonging to organic acids and derivatives were significantly negatively correlated with *R. gnavus*. Compared to the correlation between serum differential metabolites, there were more fecal differential metabolites (38 substances) that were significantly correlated with *R. gnavus*. Among them, the most noteworthy was the significant positive correlation between two secondary bile acids, chenodeoxycholic acid and isodeoxycholic acid, and *R. gnavus*. Previous studies have shown that *R. gnavus* produces 7β-Hydroxyl steroid dehydrogenases (7β-HSDHs) that convert urodeoxycholic acid (UDCA) into chenodeoxycholic acid (CDCA), thereby contributing to the formation of secondary bile acids.^42^ In fact, before and after the multidisciplinary joint intervention, the fecal levels of chenodeoxycholic acid and isodeoxycholic acid exhibited significant changes and were significantly positively correlated with all obesity phenotypes. Subsequently, we will perform KEGG enrichment analysis on the differential metabolites associated with *R. gnavus*, revealing an enrichment in the primary bile acid biosynthesis pathway. This further validates our hypothesis regarding *R. gnavus*. Based on the aforementioned results, we reasonably conclude that *R. gnavus* plays a crucial regulatory role in host bile acid metabolism during multidisciplinary joint interventions; however, it appears to impede weight loss.

### 3.5. R. gnavus *impedes weight loss in obese mice and alters gut microbiota bile acid metabolism*

#### 3.5.1. R. gnavus *impedes weight loss in obese mice*

**Figure 7.**
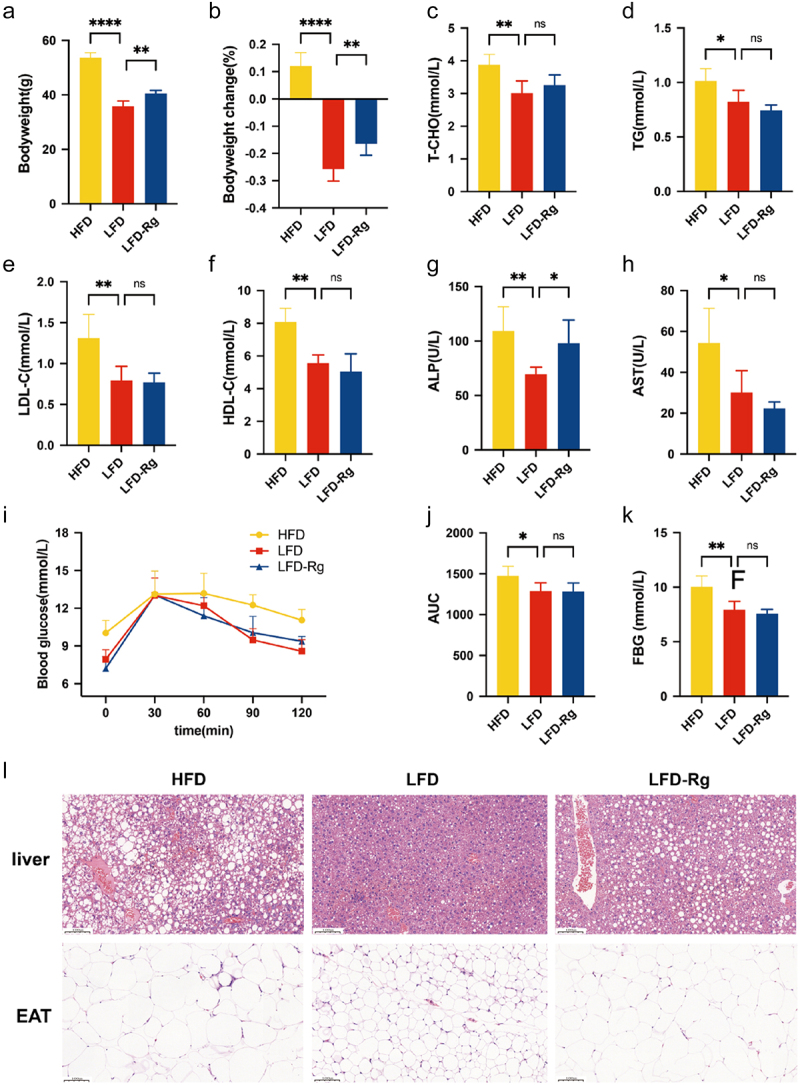
Supplementing *R. gnavus* impedes weight loss and induces liver damage in mice. a-b: mice weight status; c-f: blood lipid parameters; g-h: liver function indicators; i-k: blood glucose levels; l: liver and epididymal adipose tissue pathology in each group.

Based on the results of the MCWM in the early stages, we preliminarily believe that *R. gnavus*, a key gut symbiotic bacterium, may play a crucial role in host bile acid metabolism and energy regulation. Animal experiments were conducted to test the hypothesis. We found that the weight loss effect in mice supplemented with *R. gnavus* was less pronounced compared to mice that resumed a normal diet (Figure 7). The OGTT results showed that the AUC of the weight loss mice was significantly reduced compared to that of the mice that maintained a high-fat diet, and the fasting blood glucose was also lower than that of the high-fat group mice, whereas no significant difference in blood glucose levels were observed between the LFD-Rg and LFD groups. After the mice were sacrificed, blood lipid levels and liver function indicators were measured. The indicators of blood lipid levels and liver function in mice after weight loss were better than those in the high-fat group. *R. gnavus* supplementation did not lead to significant differences in blood lipid parameters (T-CHO, TG, LDL- C, and HDL-C) between the two groups of mice. However, the LFD-Rg group exhibited a significantly higher expression of liver injury-related ALP than the LFD group. Pathological observations of the liver appeared to corroborate this notion. In contrast to the LFD and LFD-Rg groups, the model group mice demonstrated extensive reticular voids (hepatic fat accumulation) accompanied by numerous dense circular vacuolar areas (hepatic steatosis). Nonetheless, compared with the LFD group, the liver cells of mice in the LFD-Rg group exhibited a more intensive distribution and larger volumes of fat droplets, along with a more pronounced irregular arrangement of hepatic sinusoids, suggesting liver cell damage (Figure 7). In summary, *R. gnavus* impedes weight loss and induces liver damage.

#### 3.5.2. *Supplementing* R. gnavus *alters gut microbiota*

**Figure 8.**
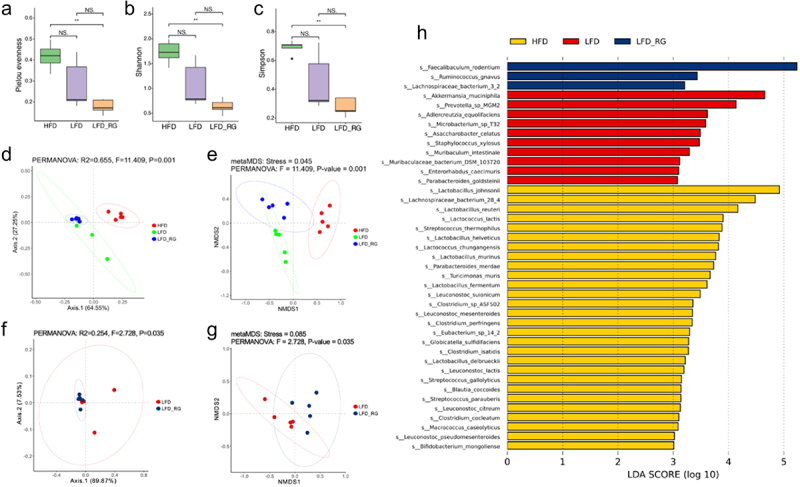
Supplementing *R. gnavus* alters gut microbiota. a-c: alpha diversity of gut microbiota; d-e: PCoA and NMDS analysis results based on bray curtis distance for the three groups of mice, respectively. f-g: PCoA and NMDS analysis results based on bray curtis distance for LFD and LFD-Rg, respectively. h: LEfSe plot showing unique species signatures identified after *R. gnavus* supplementation.

Three alpha diversities indicated that the richness and evenness of the gut microbiota in the HFD, LFD, and LFD-Rg groups gradually decreased. Although the alpha diversity of the LFD-Rg group was lower than that of the LFD group, the difference was not significant. PCoA and NMDS analyses were performed based on the bray-curtis distance. The results showed significant differences in microbial community structure among the three groups of mice. When comparing LFD and LFD-Rg groups, we observed significant differences in the microbial community structures (Figure 8). These results suggest that weight loss induces alterations in the gut microbiota, with *R. gnavus* having a substantial influence on the gut microbiota structure during weight loss. However, the mechanism by which *R. gnavus* contributes to a reduction in microbial community richness and diversity remains unclear.

The LEfSe analysis results show that, The HFD group has multiple characteristic species, including *Lactobacillus Johansonii*, *Lachnospiraceae bacteria* 28_4, *Lactobacillus reuteri*, *Lactococcus lactis*, etc (Figure 8h) . The characteristic species of the LFD group, *A. muciniphila*, *Prevotella* sp. MGM2 and *Parabacteroides goldsteinii* (*P. goldsteinii*). *A. muciniphila* is a next-generation probiotic used to prevent and treat metabolic diseases. Research has shown that *A. muciniphila* is significantly negatively correlated with physiological indicators of obesity^43^; in this study, obese mice seemed to confirm this viewpoint by controlling their diet to lose weight. Similarly, as early as 2006, scientists confirmed the weight loss effect of *P. goldsteinii* through fecal microbiota transplantation experiments, referring to it as a “lean bacteria”. Its anti-obesity effect is related to the adjustment of the composition of the gut microbiota.^44^ The characteristic species of the LFD-Rg group included *Faecalibacterium rodentium*, *R. gnavus*, *Lachnospiraceae bacterium* 3 2. The increase in *R. gnavus* abundance was largely related to supplementation with this bacterium. A recent study showed that *Faecalibacterium rodentium* can serve as a biomarker for non-obese nonalcoholic fatty liver disease,^45^ and that supplementation with *R. gnavus* increased the abundance of *Faecalibacterium rodentium* in the gut of mice, accompanied by an increase in ALP, which seems to confirm this viewpoint.

#### 3.5.3. *Supplementing* R. gnavus *alters gut microbiota metabolism*

**Figure 9.**
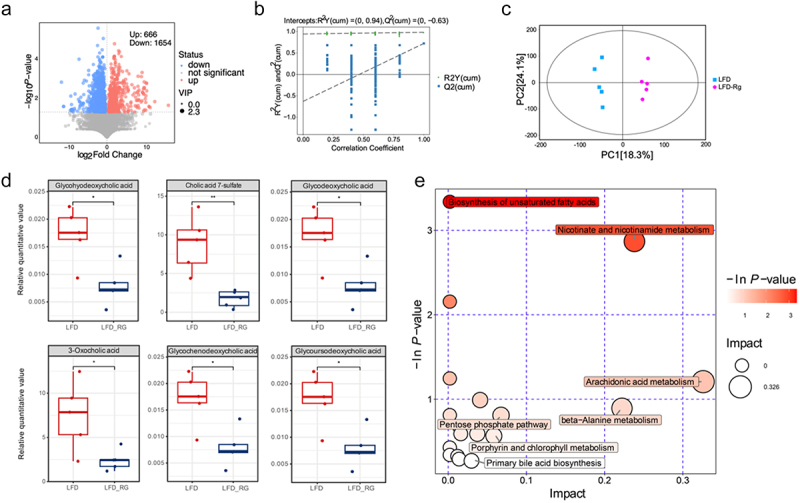
*R. gnavus* alters gut microbiota metabolism. A: volcano plot for group LFD-Rg vs LFD; B: permutation plot test of OPLS-DA model for group LFD-Rg vs LFD; C: score scatter plot of OPLS-DA model for group LFD-Rg vs LFD; D: differences in bile acids and their derivatives among differential metabolites; E:Metabolic pathway analysis for group LFD-Rg vs LFD.

We investigated the metabolic changes induced by *R. gnavus* through non-targeted metabolomics and determined whether we could obtain results similar to the association between bile acids and *R. gnavus* in the previous population cohort (Figure 9). We used OPLS-DA to compare and analyze the metabolites of LFD and LFD-Rg, and the Score scatter plot of the OPLS-DA model showed significant inter group differences in the metabolites between the two groups. The Permutation plot test of the OPLS-DA model (*n* = 200) showed Q^2^ < 0.5 and no overfitting, indicating reliable predictive performance of the model. The volcano plot shows the overall up-regulation and down-regulation of the two sets of differential metabolites. After supplementation with *R. gnavus*, 666 metabolites were significantly upregulated, and 1654 metabolites were significantly downregulated.

Surprisingly, we were pleasantly surprised discovered six types of bile acids and bile acid-binding compounds among the differential metabolites, namely cholic acid 7-sulfonate, 3-Oxocholid acid, glycoursodeoxycholic acid，glycohyodeoxycholic acid，glycodeoxycholic acid and glycochenodeoxycholic acid (Figure 9d). Although the types of bile acids identified in animal experiments differ from those associated with *R. gnavus* in previous population studies, these findings still indicate changes in bile acid composition. Pathway analysis of differential metabolites revealed highly enriched and significantly altered pathways, including primary bile acid biosynthesis. Surprisingly, the biosynthesis of primary bile acids was also enriched in the differential pathways identified in human feces. These findings confirm our previous speculation that *R. gnavus* regulates bile acid metabolism, thereby affecting energy metabolism.

## 4. Discussion

Currently, there is widespread recognition of the dangers of obesity, and efforts are being made to prevent its onset and reverse its effects. Previous studies have demonstrated a close relationship between gut microbiota and obesity, as well as related metabolic diseases. Although numerous studies have shown significant changes in gut microbiota in individuals who have successfully lost weight, many of these changes are considered accompanying states. Strategies for intervening in obesity through gut microbiota are still in their early stages, necessitating more comprehensive and in-depth exploration. Therefore, we focused on examining changes in gut microbiota characteristics and metabolic status in the population before and after MCWM weight loss. We discovered a clear association between the key bacterial strain *R. gnavus* and weight loss processes, finding evidence that it hinders weight loss in obese animal models.

Our weight loss cohort employs a “lifestyle-based” approach, integrating diet and regular exercise, with some participants also receiving medication, resulting in successful weight loss outcomes. The improvement in metabolic status is more pronounced in non-diabetic individuals compared to their diabetic counterparts. This discrepancy can be attributed to the presence of insulin resistance and chronic metabolic disorders prevalent in diabetic patients, which not only impair glucose metabolism but also disrupt lipid and protein metabolism, thereby diminishing the efficacy of weight loss interventions.^46,47^ Conversely, non-diabetic individuals generally exhibit heightened insulin sensitivity, facilitating a more efficient utilization of fats and glycogen for energy metabolism under analogous interventions, which culminates in significantly enhanced improvements across various physiological parameters.

Although significant changes were observed in the physiological parameters of the subjects (such as body weight, blood glucose, body fat percentage and lipid levels) following the intervention, no significant alterations were detected in the structure and diversity of the gut microbiota. Moreover, drug intervention appeared to have minimal impact on microbial diversity. Similar findings were reported in a study on ketogenic diet-induced weight loss.^48^ This may be attributed to the limitations of alpha and beta diversity. Jari Oksanen et al.^49^ and Lozupone et al.^50^ have noted that commonly used alpha and beta diversity primarily reflect species richness and evenness within a community. These indices capture overall community differences but do not effectively reflect community functionality, the dynamics of specific species, or sensitivity to subtle changes in species abundance. For instance, certain microbial species with important physiological functions, even if present in low abundance, may exhibit changes that are difficult to capture using these diversity indices.^51^ Therefore, it is essential to identify microbial species that show significant changes associated with physiological parameters and to explore whether these species can be considered potential biomarkers.

Previous studies have reported the identification of key microbial species involved in the weight loss process. During the initial phase of a low-calorie weight-loss diet, the abundance of *Bacteroides* increased.^52^ In obese youths from Japan^53^ and China,^54^ the abundance of *Coprococcus* was found to be high, but it decreased after laparoscopic sleeve gastrectomy^55^ and adolescent weight-loss interventions.^56^ In this study, several different species were similarly identified, which seem to support previous findings: *R. gnavus*^57^ was enriched in obese volunteers, while *O. splanchnicus*^58^ was reduced in obese children. In recent years, machine learning has been extensively used in microbiological research for classification and prediction. We applied eight machine learning classifier models to identify the key differential species. We observed that the RF classifier achieved the best classification performance while identifying the top 30 significant features that contributed to classification accuracy. Among these, recurrence of *O. splanchnicus* and *R. gnavus* was noted. These findings suggest that these two species differ in traditional analysis methods and make significant contributions to the classification outcomes of machine learning. Nevertheless, while the machine learning algorithms and statistical methods employed applied in this study are adequate for “small n, large p” problems, the limitation posed by the sample quantity may hinder the model’s ability to effectively capture potential effects; Furthermore, the uneven distribution of samples across different groups or subgroups could introduce bias in the estimation of inter-group differences. These factors may exert some impact on the analysis results.

In our study, we assessed differences at the species level as well as observed that the interaction network of the gut microbiota became more intricate post-intervention. This finding aligns with the perspective of Dugas, who in a comparative analysis of gut microbiota among 100 women from rural Ghana (50% lean, BMI < 25 kg/m^2^, and 50% obese, BMI ≥ 30 kg/m^2^), reported that leaner Ghanaian women exhibited a more cohesive and stable gut microbiota coexistence network compared to their obese counterparts.^59^ *Coprococcus eutactus*, a butyric-producing probiotic,^60,61^ was negatively correlated with *R. gnavus* before the intervention. *R. gnavus* has been reported to be positively associated with obesity and type 2 diabetes in many studies.^62^ However, after MCWM, *R. gnavus* is no longer associated with *Coprococcus eutactus*. This indicates that MCWM promotes the microbial community structure of the subjects toward a healthier state.

The MCWM significantly altered the metabolic state of the participants. After weight loss, the pathways enriched in the serum differential metabolites of the participants primarily involved amino acid metabolism, purine metabolism, and coenzyme biosynthesis, potentially due to dietary structure adjustments and changes in food intake. Research indicates that dietary interventions, such as extremely low-carbohydrate weight loss strategies, significantly alter serum metabolites in participants, with amino acids playing a significant role in differential metabolites.^63^ In addition, high levels of certain amino acids are common in patients with obesity, type 2 diabetes mellitus, or insulin resistance. Effective weight loss procedures, such as Roux-en-Y gastric bypass (RYGB), result in lower plasma amino acid levels.^64^ In the analysis of differential metabolic pathways in the feces, we observed several amino acid metabolic pathways, as well as the primary bile acid biosynthesis pathway. Bile acids can profoundly affect host metabolic and immunological functions by activating different bile acid receptors to regulate signaling pathways that control a broad range of complex symbiotic metabolic networks, including glucose, lipid and steroid metabolism, as well as modulate energy homeostasis.^65^ Host liver cells produce primary bile acids from cholesterol; once these host-derived primary bile acids enter the gastrointestinal tract, they are chemically modified into secondary bile acids by the gut microbiota. Consequently, alterations in the structure of the gut microbiota significantly influence the composition of the host bile acid pool.

We conducted correlation analyses by combining physiological indicators with metagenomic and metabolomic data to explore the potential relationships between microbiota and metabolites in the weight loss cohort. Among the differentially represented species identified using LEfSe, *R. gnavus* exhibited the strongest significant correlation with obesity-related physiological indicators. In addition, *R. gnavus* is a significant feature of the RF classifiers used in machine learning. Further analysis of the correlation between *R. gnavus* and differential metabolites in host serum and feces revealed a closer association with fecal metabolic bacteria than with serum metabolites. The reason for this is apparent: as gut symbiotic bacteria, a portion of their metabolites is directly excreted and detected in the feces. We screened for fecal differential metabolites with a significant correlation (| r |>0.1) with *R. gnavus* and found that almost all of these substances were significantly correlated with physiological indicators for evaluating obesity. Of these, two bile acids (chenodeoxycholic acid and isodeoxycholic acid) were significantly positively correlated with *R. gnavus* as well as significantly positively correlated with all obesity physiological indicators. Following the multidisciplinary intervention, the relative abundances of chenodeoxycholic acid (CDCA) and isodeoxycholic acid decreased. Previous studies have revealed that obese mice have higher fecal bile acid levels than healthy mice^66^ and that obese individuals experience increased fecal bile acid excretion post-weight loss surgery,^67^ including CDCA. Pathway analysis of differentially expressed fecal metabolites showed significant correlation (| r |>0.1) with *R. gnavus* and identified enrichment in the primary bile acid biosynthesis pathway. This pathway was also observed to undergo significant changes in the fecal metabolites in this cohort. These findings raise the question of whether *R. gnavus* influences host bile acid levels during weight loss, thereby impacting weight loss outcomes.

We investigated the role of *R. gnavus* in weight loss using animal models. Following the establishment of a high-fat diet-induced obese mouse model, a dietary intervention was implemented (administration of a low-fat diet), with one group receiving an oral supplement of *R. gnavus* suspension. After an 8-week period of weight loss, we observed that mice supplemented with *R. gnavus* experienced significantly less weight loss than those fed a low-fat diet alone. This suggests that *R. gnavus* impedes weight loss in obese mice. Wu et al. previously observed that *R. gnavus* induces abnormalities in lipid metabolism in high-fat diet-induced obese mice.^68^ However, in our dietary intervention protocol for weight loss, *R. gnavus* did not appear to lead to significant intergroup differences in blood lipid or glucose levels. This may be attributed to a period of weight loss, resulting in a decrease in body fat content to relatively normal levels in both the LFD and LFD-Rg groups. Timely dietary control prevents irreversible damage to pancreatic B-cell function. However, we were concerned regarding the significant increase in ALP levels in mice administered *R. gnavus*, which was not significantly different from that in mice consistently fed a high-fat diet, indicating liver damage. In addition, evidence of liver cell damage in the LFD-Rg mice was directly observed by pathological examination of the liver.

Supplementation with *R. gnavus* significantly altered the gut microbiota. We observed a significant reduction in alpha diversity following supplementation, potentially due to the inhibitory effects of *R. gnavus* metabolites on other gut symbiotic bacteria. Previous studies have reported that upon colonization in rats, *R. gnavus* produces an antimicrobial peptide that is as effective against the pathogen *Clostridium perfringens* as metronidazole.^69^ We found that the abundance of *Faecalibaculum rodentium* increased following supplementation with *Faecalibaculum rodentium*, serving as a gut microbiota marker for non-obese nonalcoholic fatty liver disease.^45^ Concurrently, we observed an increase in ALP and liver tissue damage in the LFD-Rg group, which appeared to be an evidence of liver injury. In the LFD group, the mice were enriched with *A. muciniphila* and *P. goldsteinii. A. muciniphila*, known as the “next-generation probiotic”,^70^ is used for the prevention or treatment of metabolic diseases.^71^ Research indicates that *A. muciniphila* exhibits a significant negative correlation with obesity-related physiological indicators, and the weight loss observed in obese mice with dietary control in this study further supports this hypothesis. Similarly, as early as 2006, scientists demonstrated the weight loss efficacy of *P. goldsteinii* through fecal microbiota transplantation experiments, dubbing it the “thin bacteria.” Its anti-obesity effects are associated with alterations in the gut microbiota composition.^44,72^

We conducted a comparative analysis of fecal metabolites in mice fed a control diet (LFD) and a treatment diet (LFD-Rg) using non-targeted metabolomics to identify significant differences between the two groups. The differential metabolites included six bile acids and their derivatives, and pathway analysis indicated significant enrichment in the primary bile acid biosynthesis pathway, mirroring previous findings across various scientific cohorts. Research has shown that *R. gnavus* possesses a hydroxysteroid dehydrogenase (HSDH) that converts bile acids into isomers known as iso-bile acids.^73^ These iso-bile acids can activate the farnesoid X receptor (FXR) in collaboration with endogenous ligands,^74^ thereby precisely regulating the synthesis and transport of bile acids. This likely accounts for the alterations in the primary bile acid biosynthesis pathway observed following *R. gnavus* supplementation.

Based on both the population analysis and animal experimental results, we can infer that *R. gnavus*, a prominent characteristic species in obese subjects in this study, impairs weight loss, harms the liver, and alters bile acid metabolism. However, further investigation is required to ascertain whether the alterations in bile acid metabolism induced by *R. gnavus* are pivotal for influencing weight loss. Numerous studies have been conducted on gut biomarkers of obesity; however, their scientific validity and efficacy require verification. If we can clearly elucidate the mechanisms by which certain biomarkers regulate energy metabolism, this will significantly contribute to advancing the health management industry focused on the gut microbiota.

## 5. Conclusions

We identified *R. gnavus* as a potential biomarker of obesity in a cohort study of the MCWM for weight loss. Its abundance significantly decreased at 6 months post-intervention and was positively correlated with multiple obesity-related clinical indicators. Two types of bile acids significantly correlated with *R. gnavus*, and differential analysis of the metabolite pathways associated with *R. gnavus* revealed alterations in the bile acid metabolism pathway. Animal experiments have confirmed that *R. gnavus* impedes weight loss achieved through dietary regulation, leading to liver tissue damage and altered bile acid metabolism.

## Supplementary Material

Supplementary materials_revised.docx
