# Supplementary material for: Microbial perspective of multidisciplinary collaborative weight management approach: Ruminococcus gnavus may serve as a key target for weight loss
Source: Gut Microbes. 2024 Dec 31;17(1):2442038. doi: 10.1080/19490976.2024.2442038 (PMC12931726; doi:10.1080/19490976.2024.2442038)
Supplement: Supplementary materials_revised.docx [file KGMI_A_2442038_SM1580.docx]

Figure S1


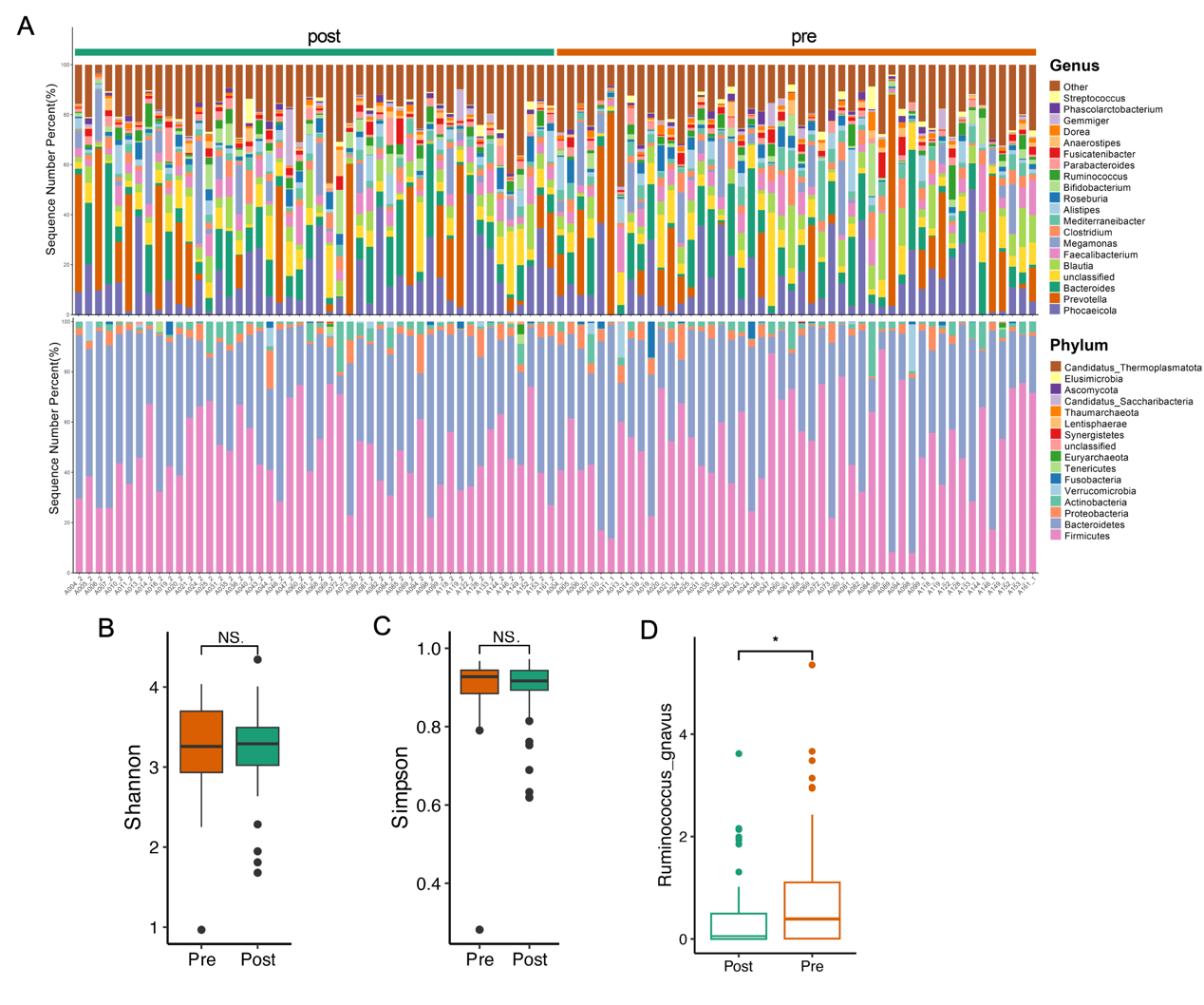


Figure S1. Analysis of Gut Microbiota Composition and Changes Pre- and Post- MCWM. A: Stacked bar charts showing the relative abundance of gut microbiota at the genus and phylum levels pre- and post- MCWM. B-C: Box plot of Shannon diversity index and Simpson diversity index comparing pre- and post- MCWM. D: The abundance of *R. gnavus* pre- and post- MCWM.

Figure S2


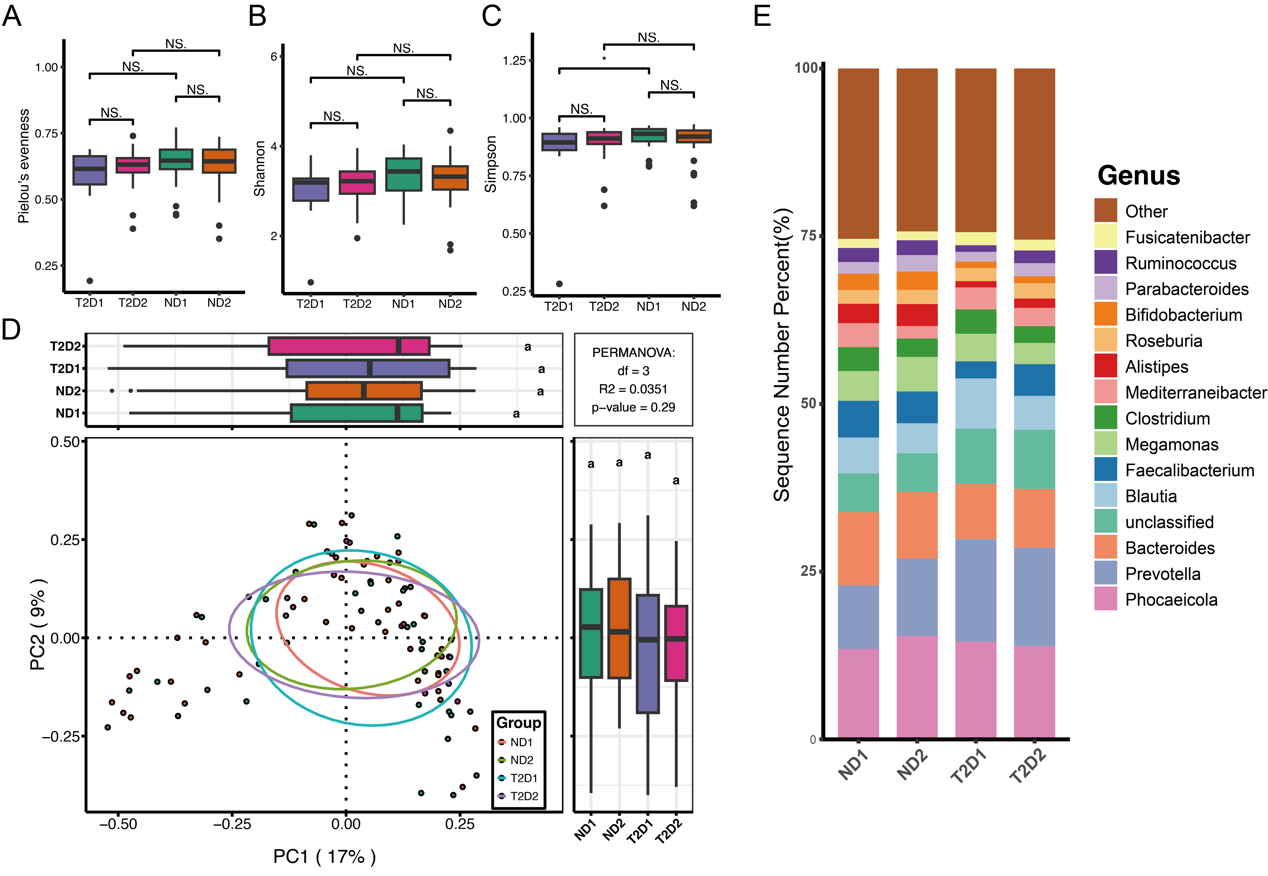


Figure S2. The effects of the MCWC intervention on the gut microbiota diversity and composition in diabetic (T2D1, T2D2) and non-diabetic (ND1, ND2) subjects. T2D1: Diabetic patients before the MCWC intervention. T2D2: Diabetic patients after the intervention. ND1: Non-diabetic subjects before the intervention. ND2: Non-diabetic subjects after the intervention. A-C: Alpha diversity metrics (A: Pielou's evenness, B: Shannon index, C: Simpson index) showing gut microbiota diversity across the four groups. D: Beta diversity comparison using principal coordinates analysis (PCoA) based on Bray-Curtis distance. Each point represents the microbial community structure of an individual sample. The ellipses represent 95% confidence intervals for each group. PERMANOVA analysis indicates no significant differences in beta diversity between the groups (p = 0.29). E: Bar plot showing the relative abundance of bacterial genera across the four groups.

Figure S3


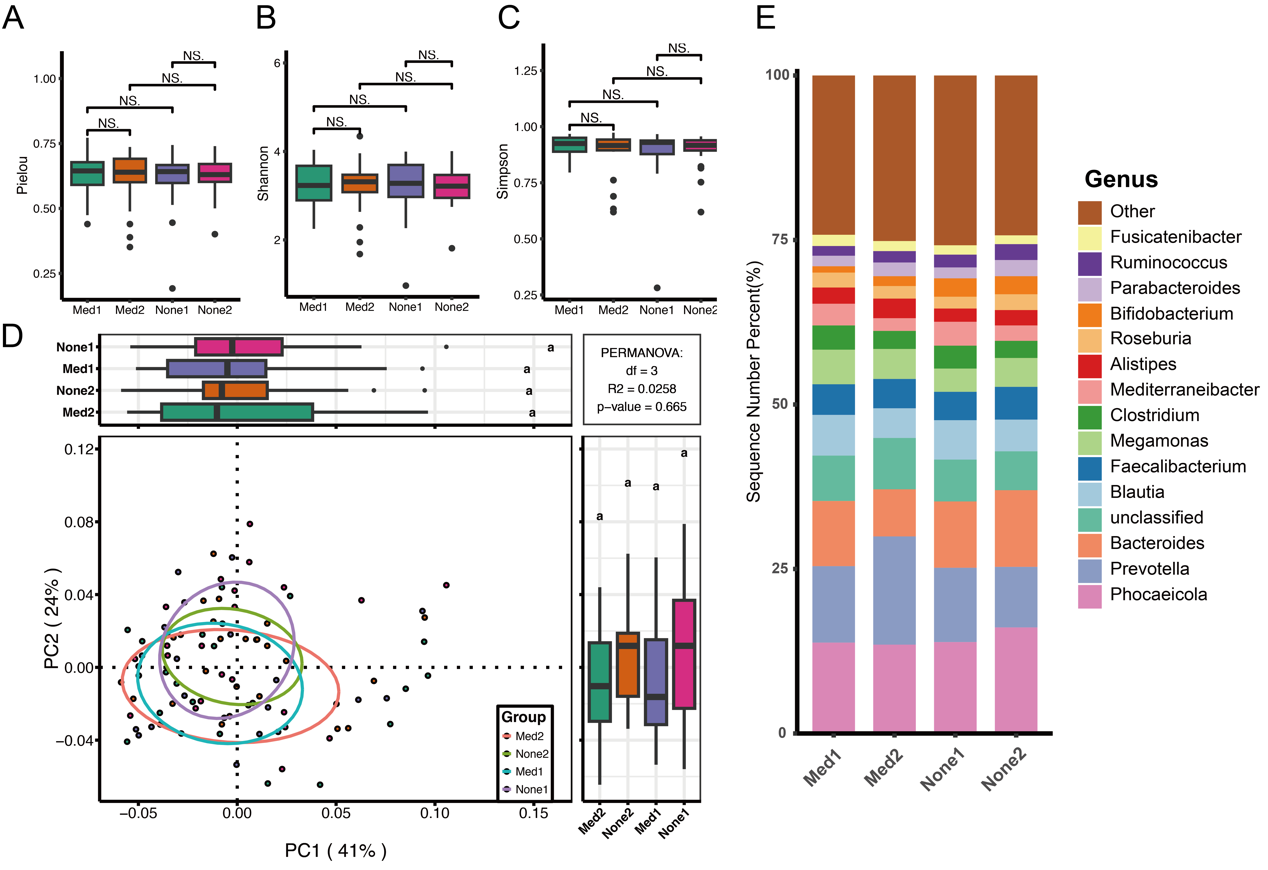


Figure S3. The effects of the MCWC intervention on the gut microbiota diversity and composition in medicated (Med1, Med2) and non-medicated (None1, None2) subjects. Med1: Medicated subjects before the MCWC intervention. Med2: Medicated subjects after the intervention. None1: Non-medicated subjects before the intervention. None2: Non-medicated subjects after the intervention. A-C: Alpha diversity metrics (A: Pielou's evenness, B: Shannon index, C: Simpson index) showing gut microbiota diversity across the four groups. D: Beta diversity comparison using principal coordinates analysis (PCoA) based on Bray-Curtis distance. Each point represents the microbial community structure of an individual sample. The ellipses represent 95% confidence intervals for each group. PERMANOVA analysis indicates no significant differences in beta diversity between the groups (p = 0.665). E: Bar plot showing the relative abundance of bacterial genera across the four groups.

Figure S4


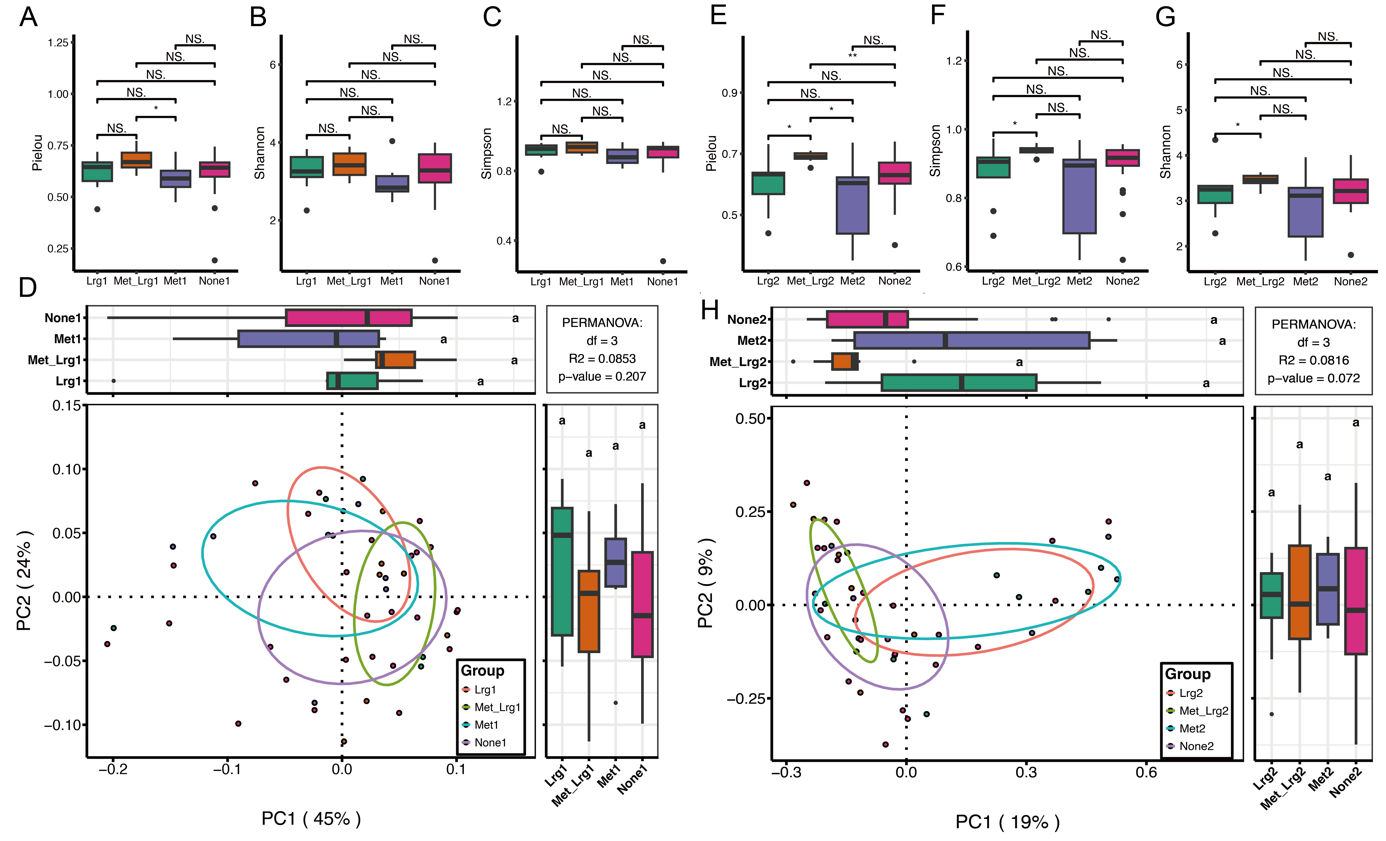


Figure S4. Analysis of gut microbiota structure in patients under different medication regimens (metformin alone, liraglutide alone, combined metformin and liraglutide, and no medication). A-C: Baseline microbial diversity indices, including Pielou's evenness, Simpson, and Shannon indexes, among the four groups. D: Principal Coordinates Analysis (PCoA) plot showing baseline microbiota composition. E-G: Post-intervention microbial diversity indices for each group. H: PCoA plot depicting the post-intervention microbiota structure.

Figure S5

**
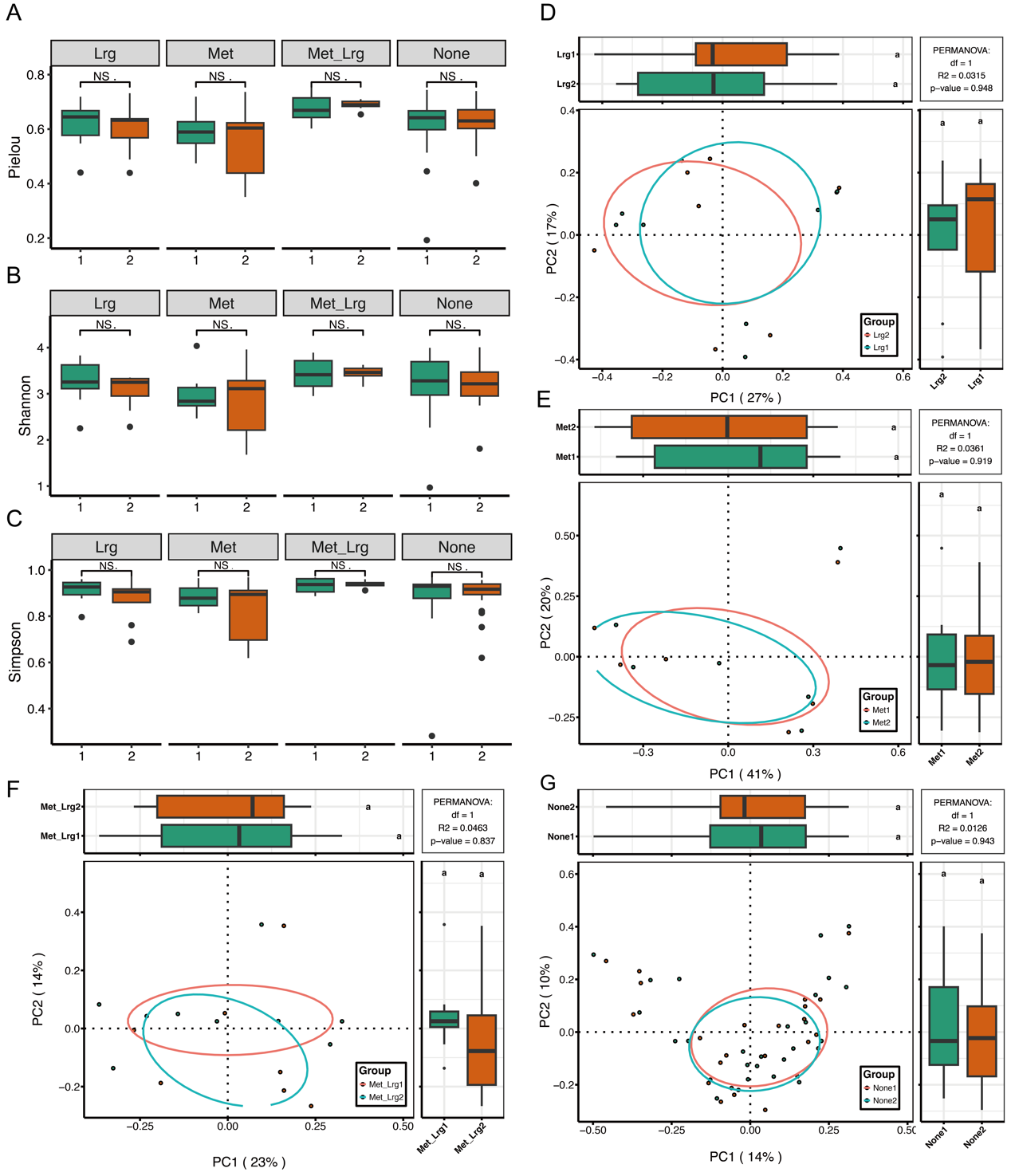
**

Figure S5. Comparison of changes in gut microbiota structure before and after intervention for each medication regimen (liraglutide alone, metformin alone, combined metformin and liraglutide, and no medication). A-C: Alpha diversity indices (Pielou's evenness, Shannon index, and Simpson index) showing no significant changes in microbial diversity before and after intervention within each group. D-G: Principal Coordinates Analysis (PCoA) plots illustrating the beta diversity of gut microbiota, indicating no significant shifts in microbial composition across all medication regimens.

Figure S6


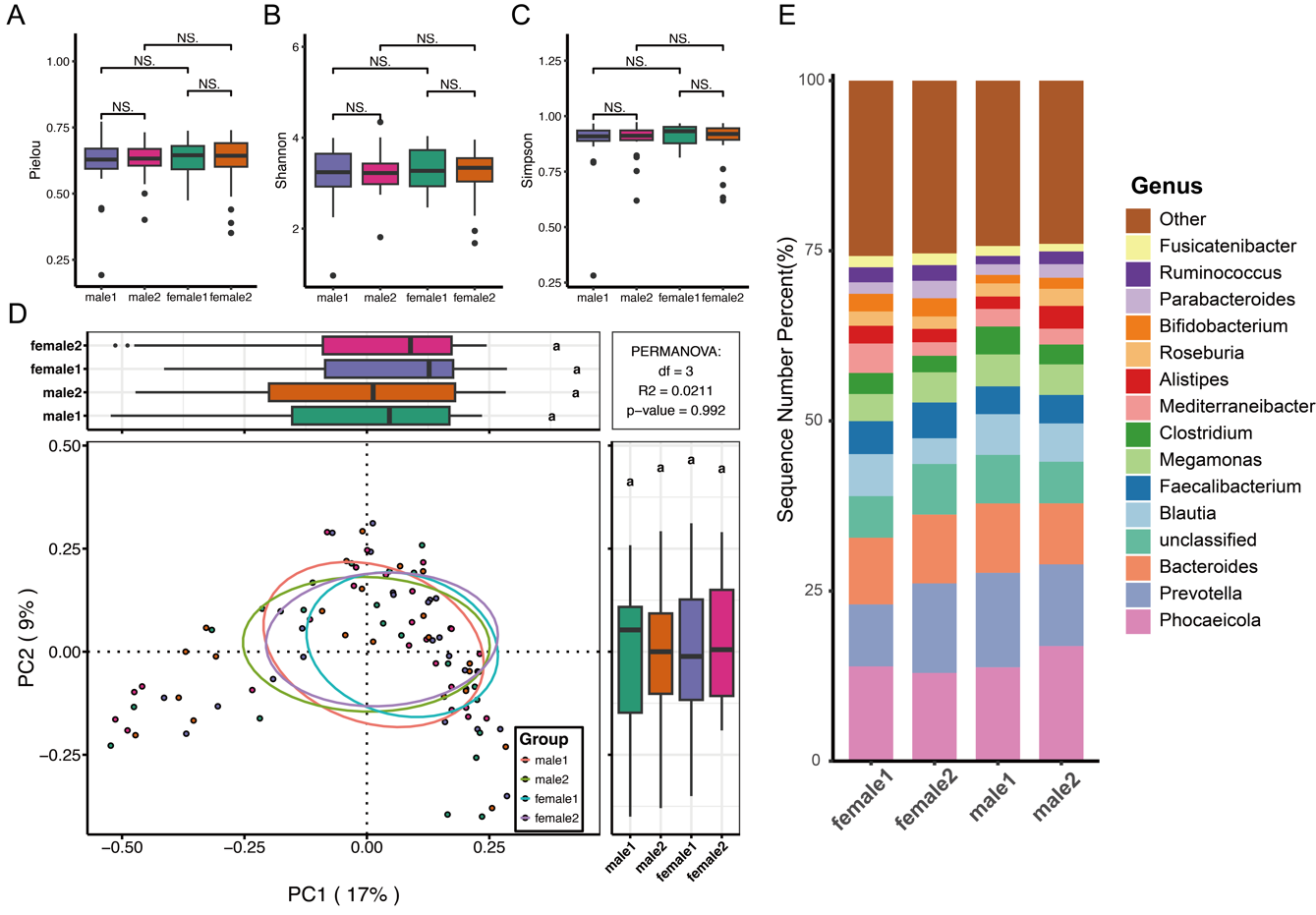


Figure S6. The effects of the MCWC intervention on the gut microbiota diversity and composition in male (male1, male2) and female (female1, female2) subjects. male1: Male subjects before the MCWC intervention. male2: Male subjects after the intervention. female1: Female subjects before the intervention. female2: Female subjects after the intervention. A-C: Alpha diversity metrics (A: Pielou's evenness, B: Shannon index, C: Simpson index) showing gut microbiota diversity across the four groups. D: Beta diversity comparison using principal coordinates analysis (PCoA) based on Bray-Curtis distance. Each point represents the microbial community structure of an individual sample. The ellipses represent 95% confidence intervals for each group. PERMANOVA analysis indicates no significant differences in beta diversity between the groups (p = 0.992). E: Bar plot showing the relative abundance of bacterial genera across the four groups.

Figure S7


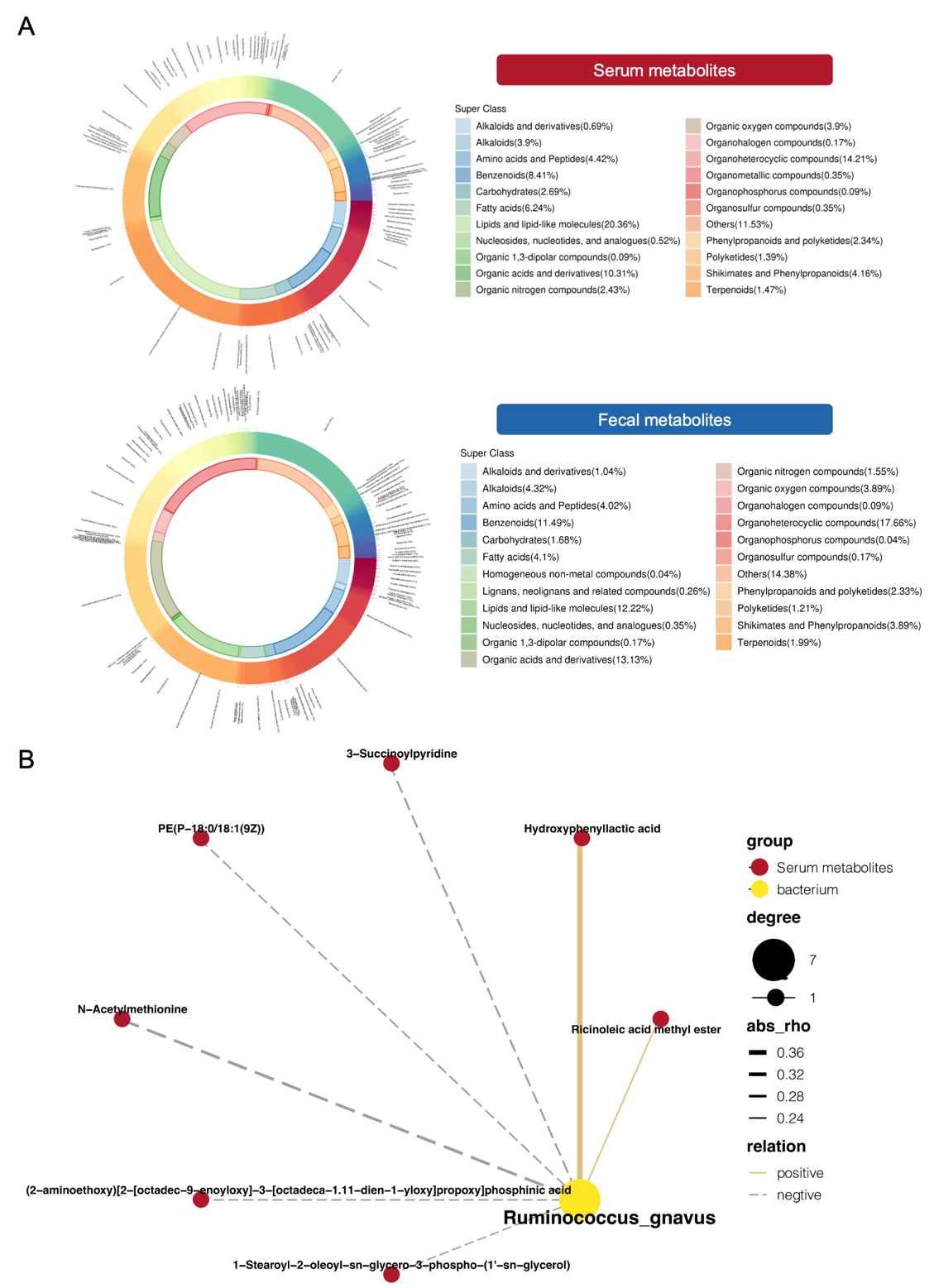


Figure S7. Classification of Serum and Fecal Metabolites and Correlation of Serum Metabolites with *R. gnavus.* A: Superclass classification of serum and fecal metabolites. Different colors represent different superclasses, with the percentage of each superclass indicated in parentheses. B: Correlation network of *R. gnavus* with serum metabolites. Yellow nodes represent *R. gnavus*, red nodes represent serum metabolites, solid lines indicate positive correlations, and dashed lines indicate negative correlations. The thickness of the lines is proportional to the absolute value of the correlation coefficient (abs_rho).

Table S1

Table S1 Body composition of subjects

| Index | Pre intervention | Post intervention | *P* | Change | Change rate |
| --- | --- | --- | --- | --- | --- |
| SM [kg] | 33.625±7.207 | 32.475±7.608 | 0.831 | -1.150±1.946 | -3.641±6.203% |
| BF [kg] | 40.786±10.209 | 30.932±10.699 | <0.001 | -9.855±6.531 | -24.294±14.239% |
| PBF (%) | 40.561±6.098 | 34.530±7.081 | <0.001 | -6.032±4.244 | -15.016±9.716% |
| BMR [kcal/day] | 1656.273±256.797 | 1595.977±304.941 | 0.607 | -60.295±151.618 | -3.756±9.359% |

The table presents the body composition parameters of obese subjects, including skeletal muscle mass (SM), body fat mass (BF), body fat percentage (PBF), and basal metabolic rate (BMR), measured pre- and post-intervention. The values are expressed as mean ± standard deviation. The change and change rate were calculated based on the difference between the post- and pre-intervention values. P-values were derived from analysis of covariance (ANCOVA), with adjustments for gender and age as covariates.

Table S2

Table S2 Clinical parameters of diabetes subjects and non-diabetic subjects

| Index | Diabetes subjects | | | Non-diabetic subjects | | |
| --- | --- | --- | --- | --- | --- | --- |
|  | Pre intervention | Post intervention | *P* | Pre intervention | Post intervention | *P* |
| BW [kg] | 101.731±20.760 | 89.333±21.284 | <0.001 | 100.203±16.164 | 88.606±17.163 | <0.001 |
| BMI [kg/m^2^] | 34.426±4.671 | 30.268±4.895 | <0.001 | 35.546±4.773 | 31.408±5.433 | <0.001 |
| WC (cm) | 108.050±12.541 | 96.700±12.837 | <0.001 | 109.661±10.550 | 97.310±14.857 | <0.001 |
| HC (cm) | 111.938±9.407 | 105.433±10.112 | <0.001 | 114.694±9.039 | 106.145±11.898 | <0.001 |
| WHR | 0.965±0.063 | 0.915±0.052 | <0.001 | 0.957±0.069 | 0.914±0.072 | 0.008 |
| SBP [mmHg] | 132.500±17.139 | 121.867±13.103 | 0.001 | 135.067±14.391 | 122.419±14.440 | <0.001 |
| DBP [mmHg] | 79.188±13.136 | 69.000±7.810 | 0.001 | 80.300±9.326 | 71.290±9.758 | <0.001 |
| ALT [U/L] | 73.850±49.220 | 60.213±82.704 | 0.407 | 69.265±55.595 | 28.874±21.235 | 0.008 |
| AST [U/L] | 46.013±25.196 | 31.467±21.216 | 0.139 | 42.442±27.385 | 22.097±13.095 | 0.004 |
| ALP [U/L] | 93.556±29.006 | 52.953±16.220 | <0.001 | 80.129±19.988 | 52.403±12.765 | <0.001 |
| ALB [g/L] | 45.163±2.492 | 83.960±79.226 | 0.410 | 45.539±6.225 | 67.087±20.464 | <0.001 |
| BA [µmol/L] | 4.193±2.827 | 2.871±1.369 | 0.255 | 3.829±2.785 | 2.406±1.166 | 0.127 |
| GGT [U/L] | 60.538±34.341 | 60.467±107.035 | 0.874 | 47.042±24.150 | 41.848±58.863 | 0.905 |
| CREA [µmol/L] | 69.513±14.262 | 74.273±12.594 | <0.001 | 65.419±17.701 | 68.210±15.803 | <0.001 |
| BUN [mmol/L] | 5.208±1.271 | 5.320±1.399 | 0.277 | 4.210±1.239 | 5.042±0.965 | 0.006 |
| UA [µmol/L] | 424.669±138.539 | 415.040±81.517 | 0.323 | 437.777±129.542 | 424.171±117.322 | 0.147 |
| TG [mmol/L] | 2.796±1.575 | 1.824±0.912 | 0.013 | 2.133±0.987 | 1.622±0.994 | 0.237 |
| TC [mmol/L] | 5.339±1.402 | 4.908±1.003 | 0.645 | 5.202±0.844 | 5.053±0.915 | 0.353 |
| LDL-C [mmol/L] | 3.294±0.911 | 2.919±0.740 | 0.711 | 3.210±0.631 | 3.027±0.767 | 0.753 |
| HDL-C [mmol/L] | 1.005±0.137 | 1.055±0.137 | 0.002 | 1.027±0.189 | 1.088±0.199 | 0.001 |
| HbAlc [%] | 6.296±1.743 | 5.267±0.431 | 0.313 | 6.861±2.056 | 5.424±0.359 | 0.020 |
| FBG [mmol/L] | 6.481±3.087 | 5.277±0.581 | 0.451 | 6.496±2.663 | 5.097±0.629 | 0.061 |
| BG2h [mmol/L] | 9.503±5.981 | 6.820±2.059 | 0.228 | 10.409±4.793 | 7.040±1.780 | <0.001 |
| INS0h [μIU/ml] | 33.678±18.114 | 22.571±22.019 | <0.001 | 28.524±17.782 | 18.570±12.938 | <0.001 |
| INS2h [μIU/ml] | 158.378±138.423 | 104.530±130.930 | 0.040 | 121.330±85.236 | 75.867±61.528 | 0.032 |

The table presents the body composition parameters of diabetes subjects and non-diabetic subjects, including body weight(BW), body mass index(BMI); waist circumference(WC), hip circumference(HC), waist to hip ratio(WHR), systolic blood pressure(SBP), diastolic blood pressure(DBP), alanine transferase(ALT), aspartate transaminase(AST), alkaline phosphatase(ALP), bile acid(BA), gamma-glutamyl transpeptidase(GGT), creatinine(CREA), blood urea nitrogen(BUN), uric acid(UA), triglyceride(TG), total cholesterol (TC), low-density lipoprotein cholesterol(LDL -C), high density liptein cholesterol(HDL -C); haemoglobin Alc(HbA1c), fasting blood glucose (FBG), 2-hour blood glucose(BG2h), fasting insulin(INS0h) and 2-hour insulin(INS2h)measured pre- and post-intervention. P-values were derived from analysis of covariance (ANCOVA), with adjustments for sex, age, height as covariates.

Table S3

Table S3 Coefficients and p-values from linear regression models on diversity indices

| Index | Lrg  Coef | Lrg  P-value | Met Coef | Met  P-value | Met_Lrg Coef | Met_Lrg P -value | age  Coef | age  P -value | gender Coef | gender  P -value |
| --- | --- | --- | --- | --- | --- | --- | --- | --- | --- | --- |
| Shannon | 0.0463 | 0.8322 | -0.3836 | 0.1212 | 0.2390 | 0.2836 | -0.0062 | 0.5584 | 0.004 | 0.9799 |
| Simpson | -0.0252 | 0.4838 | -0.0759 | 0.0637 | 0.0467 | 0.2040 | -0.0013 | 0.4517 | 0.0001 | 0.9973 |
| Pielou | -0.0239 | 0.4928 | -0.0724 | 0.0674 | 0.0699 | 0.0521 | -0.0011 | 0.5185 | 0.0069 | 0.7870 |

Multiple linear regression models that analyze the effects of different medication statuses (liraglutide alone (Lrg), metformin alone (Met), combined metformin and liraglutide (Met_Lrg)), age, and gender on microbial diversity indices (Shannon, Simpson, and Pielou). Coefficients (Coef) represent the strength and direction of the association, while p-values assess the statistical significance. P-value below 0.05 indicates statistical significance. The "None" group (no medication) serves as the control and is not shown in the table.

Table S4

Table S4 Random forest classifier performance metrics

| Parameter | Performance metrics | Standardized_Data |
| --- | --- | --- |
| n_estimators = 400 max_depth = 15 min_samples_leaf = 2 min_samples_split = 2 | Accuracy | 0.55 |
|  | Precision | 0.67 |
|  | OOB Error | 0.48 |
|  | Confusion Matrix | [5 3]  [6 6] |

Performance metrics of the random forest classifier based on standardized data. Key parameters used in the random forest model (number of trees, depth, minimum samples required for leaf nodes and node splitting) along with performance metrics such as accuracy, precision, out-of-bag error, and confusion matrix results.
